# Mucin *O*-glycan-microbiota axis orchestrates gut homeostasis in a diarrheal pig model

**DOI:** 10.1186/s40168-022-01326-8

**Published:** 2022-08-31

**Authors:** Bing Xia, Ruqing Zhong, Weida Wu, Chengzeng Luo, Qingshi Meng, Qingtao Gao, Yong Zhao, Liang Chen, Sheng Zhang, Xin Zhao, Hongfu Zhang

**Affiliations:** 1 State Key Laboratory of Animal Nutrition, Institute of Animal Science, Chinese Academy of Agricultural Sciences, Beijing, 100193 China; 2 Animal Science and Technology College, Beijing University of Agriculture, Beijing, 102206 China; 3 Institute of Biotechnology, Cornell University, Ithaca, NY 14853 USA; 4 Department of Animal Science, McGill University, Montreal, Quebec H9X3V9 Canada

**Keywords:** Gut microbiota, Mucus layer, Mucin *O*-glycans, Glycan-microbiota interaction, Pig model

## Abstract

**Background:**

Post-weaning diarrhea in piglets reduces growth performance and increases mortality, thereby causing serious economic losses. The intestinal epithelial cells and microbiota reciprocally regulate each other in order to maintain intestinal homeostasis and control inflammation. However, a relative paucity of research has been focused on the host-derived regulatory network that controls mucin *O*-glycans and thereby changes gut microbiota during diarrhea in infancy. At the development stage just after birth, the ontogeny of intestinal epithelium, immune system, and gut microbiota appear similar in piglets and human infants. Here, we investigated the changes of mucin *O*-glycans associated with gut microbiota using a diarrheal post-weaned piglet model.

**Results:**

We found that diarrhea disrupted the colonic mucus layer and caused aberrant mucin *O*-glycans, including reduced acidic glycans and truncated glycans, leading to an impaired gut microenvironment. Subsequently, the onset of diarrhea, changes in microbiota and bacterial translocation, resulting in compromised epithelial barrier integrity, enhanced susceptibility to inflammation, and mild growth faltering. Furthermore, we found the activation of NLRP3 inflammasome complexes in the diarrheal piglets when compared to the healthy counterparts, triggered the release of proinflammatory cytokines IL-1β and IL-18, and diminished autophagosome formation, specifically the defective conversion of LC3A/B I into LC3A/B II and the accumulation of p62. Additionally, selective blocking of the autophagy pathway by 3-MA led to the reduction in goblet cell-specific gene transcript levels in vitro.

**Conclusions:**

We observed that diarrheal piglets exhibited colonic microbiota dysbiosis and mucosal barrier dysfunction. Our data demonstrated that diarrhea resulted in the activation of inflammasomes and autophagy restriction along with aberrant mucin *O*-glycans including reduced acidic glycans and truncated glycans. The results suggested the mucin O-glycans-microbiota axis is likely associated with diarrheal pathogenesis. Our study provides novel insights into the pathophysiology of early-weaning-induced diarrheal disease in piglets and potentially understanding of disease mechanisms of diarrhea for human infants. Understanding the molecular pathology and pathogenesis of diarrhea is a prerequisite for the development of novel and effective therapies. Our data suggest that facilitating *O*-glycan elongation, modifying the microbiota, and developing specific inhibitors to some key inflammasomes could be the options for therapy of diarrhea including human infants.

**Graphical abstract:**

Video abstract

**Supplementary Information:**

The online version contains supplementary material available at 10.1186/s40168-022-01326-8.

## Background

The mucus layer, which is the first line of defense limiting the translocation of potentially harmful antigens, and microbiota reciprocally regulate each other in order to maintain intestinal homeostasis and control inflammation [1]. The colonic mucus layer is produced by goblet cells and comprised of two discrete layers. The inner mucus layer is firmly attached to the epithelium to help prevent against bacterial invasion, while the outer mucus layer, a loose matrix structure, serves as the habitat for the bacteria [2]. Secretion of mucin from the goblet cells is governed by inflammasome signaling and autophagy [3, 4]. Mucus is a large polymeric network of mucins that are heavily *O*-glycosylated and O-glycans typically make up more than 80% of the mass of a mucin [5]. *O*-glycan is primarily composed of *N*-acetyl-galactosamine (GalNAc), *N*-acetyl-glucosamine (GlcNAc), fucose, galactose, mannose, and sialic acid [6]. Mucin *O*-glycans are built upon an GalNAc that is *O*-linked to serine/threonine residues. The GalNAcα-Ser/Thr structure forms the Tn antigen, which is the substrate for glycosyltransferases that further elongate into core 1 and core 3 structures in the intestine [6]. Core 1 structure is formed by the addition of galactose to the Tn antigen via the enzyme core 1 β1,3-galactosyltransferase. Biosynthesis of core 3 structure is likewise initiated by core 3 β1,3N-acetylglucosaminyltransferase transferring GlcNAc to the Tn antigen [7]. These structures can be further elongated with GlcNAc to form core 2 from core 1 and core 4 from core 3. Mucin-derived *O*-glycans play a critical role in intestinal barrier function by supplying attachment sites [8], serving as a source of nutrients for specific bacteria [9], and protecting the mucus layer from bacterial protease degradation [10]. Excessive glycan degradation by bacteria leads to erosion of the mucus layer, dysfunction of the intestinal barrier, as well as increased host susceptibility to pathogens, and inflammation [11]. Notably, loss of core 1 and core 3 *O*-glycans led to the breach of colonic mucus barrier and severe spontaneous chronic colitis, which is concurrent with microbiota-mediated activation of Caspase 1- and 11-dependent inflammasomes [12].

Using glycans as an energy source, the microbiota relies on the glycoside hydrolases generating short chain fatty acids (SCFAs) [13], which in turn, promote secretion of mucin and maintain epithelial barrier integrity [14]. This yields a mutualist relationship between glycans and the gut microbiota. The microbiota is shaped by host genetics and environmental factors [15] and is unstable during infancy [16]. The adverse effects of gut microbiota dysbiosis on host health have long been appreciated. Recent studies have reported that colonic microbial dysbiosis is associated with mucosal immune dysfunction in infant rhesus macaques during active diarrhea [17] and in mice exposed to chronic stress [18]. A study on stressed mice with diarrheal phenotype further suggested that microbial dysbiosis triggers bacterial translocation and colonic barrier dysfunction via the opening of goblet cell-associated passages, subsequently enhancing IgA responses to commensal bacteria [19]. In addition, the microbiota-derived metabolites, including bile acids (BAs), SCFAs, and tryptophan metabolites, are agents of microbe-host communication network, which is essential for maintaining host physiology [20]. Butyrate is the preferred energy source for colonocytes [21] and promotes epithelial homeostasis via activation of inflammasome-IL-18 axis with other SCFAs [22]. It should be noted that secondary bile acids (SBA) are also essential metabolites that are transformed from primary bile acids (PBA) by gut microbiota [20] and ameliorate inflammation in three murine colitis models [23]. Inflammatory bowel disease (IBD) patients had lower SBA and higher conjugated bile acid concentrations compared with healthy subjects and showed broad impairments in transformation, deconjugation, and desulfation of bile acids [24, 25].

The practice of early weaning of piglets between 14 and 30 days of age can shorten the slaughter cycle of pigs and improve reproductive performance in sows [26]. However, early weaned piglets are prone to develop diarrhea. Post-weaning diarrhea reduces growth performance and increases mortality, thereby causing serious losses [27]. Despite the progress on substantial reductions in diarrhea mortality achieved over the past decades, diarrhea remains a leading cause of childhood morbidity and mortality globally [28]. Over 400,000 children under 5 years of ages died of diarrheal disease in 2019 [29]. In addition to high mortality rates, diarrhea in early life results in a vicious feedback loop of malnutrition [30] and gut damage, including physiological and functional changes [31], which ultimately leads to growth deficit [32]. However, studies on human infants with diarrheal and intestinal diseases unavoidably suffer from limitation of fecal sample analysis only, instead of the microbiome data generated from the intestinal tract sections. A deeper understanding of the pleiotropic impact of diarrhea on gastrointestinal tract is a key to develop potential targeted treatments. Consequently, the pig may be an appropriate model for the study of gastrointestinal diseases given the anatomical and physiological similarities in neonatal piglets and human infants [33]. Previous studies have showed that IBD patients were associated with an increased abundance of truncated *O*-glycans, reduction of several complex glycans [34], and reduced terminal sulfation in the colon [35]. These significant changes in the *O*-glycans correlated with both the degree of inflammation documented in the biopsies and the severity of disease course [34]. However, the correlation of diarrheal disease to abnormal mucin *O*-glycans is unknown.

In this study, we investigate the relationship between microbial community, mucin *O*-glycans, and its impact on gut homeostasis during diarrhea, with data from both in vivo and vitro experiments. We hypothesized that diarrheal disease could cause aberrant mucin *O*-glycosylation profiles, gut microbiome dysbiosis, and compromised mucosal barrier function, leading to an impaired gut microenvironment.

## Results

### Diarrheal piglets show colonic microenvironment dysbiosis

In this study, piglets were weaned early from their sows at 21 days of age. Eight piglets (28 days old) with active diarrhea were selected from 6 l. Gender-matched littermates were used as the healthy controls. The overall structure of mucosal and luminal microbiota in the colon investigated by an UniFrac-based principal coordinates analysis (PCoA) showed that diarrheal piglets displayed a shift clustering of bacterial composition, which was distinct from healthy controls (Fig. S1 A-B). There was no significant difference in the mucosal and luminal α-diversity, except for the mucosal Shannon index which trended declined (Fig. S1 C-J). A linear discriminant analysis effect size (LEfSe) analysis was employed to identify differentially abundant bacterial taxa in the diarrheal pigs and healthy controls (Fig.S1 K-L, Table S1). In the mucosa, diarrheal piglets also had significantly higher proportions of the genera *Lactobacillus*, *Peptococcus*, *Campylobacter*, and *Peptostreptococcus*. Many of the genera, *Phascolarctobacterium*, *Lachnospiraceae FCS020 group*, and *Eubacterium hallii group*, from *Firmicutes* were also enriched in the colonic mucosa of healthy controls. In the colonic lumen, diarrheal piglets presented markedly greater proportions of the genera *Lactobacillus*, *Escherichia Shigella*, *Anaerobiospirillum*, *Peptostreptococcus*, and *Alloprevotella* than healthy controls did. Moreover, many taxa, *Gammaproteobacteria*, *Enterobacteriaceae*, and *Enterobacterales* from phyla *Proteobacteria* were found to be significantly elevated in the diarrheal piglets.

Having demonstrated the colonic microbiota dysbiosis in diarrheal piglets, we next sought to examine the alteration of bacteria-derived metabolites. We observed substantially lower concentrations of butyrate in both the colonic contents and feces of the diarrheal piglets (Fig. S2 E). By contrast, the level of lactate was significantly increased in the diarrheal piglets (Fig. S2 H). Bile acid metabolism is one of the key metabolic pathways tremendously affected by the gut microbiota changes [36]. A targeted metabolomic profiling by liquid chromatography-tandem mass spectrometry (LC-MS/MS) was conducted to assess the differences in the bile acid composition of colonic contents and feces from diarrheal piglets versus the healthy controls (Fig. S2 I-J). The most striking difference in the bile acid profiling analysis was that two SBAs, lithocholic acid (LCA) and hyodeoxycholic acid (HDCA), were significantly reduced in the feces of diarrheal piglets compared with the healthy controls (Fig. S2 L, Fig. S3 E-F). Fecal deoxycholic acid (DCA) level was also marginally lower in diarrheal piglets than the healthy controls, although this was not significant (Fig. S3 D). Conversely, diarrheal piglets exhibited a significantly higher cholic acid (CA) concentration in the colonic content (Fig. S3 A).

Compared with the healthy controls, diarrheal piglets displayed a tendency towards decreased body weight (Fig. S4 A). No significant changes were observed in the entire intestine and colon length of diarrheal piglets (Fig. S4 B-D). However, the liver and spleen weight were significantly higher in pigs with diarrhea (Fig. S4 E-H).

### Piglets with diarrhea exhibit a breached inner mucus layer and aberrant mucin O-glycan profile

**Fig. 1:**
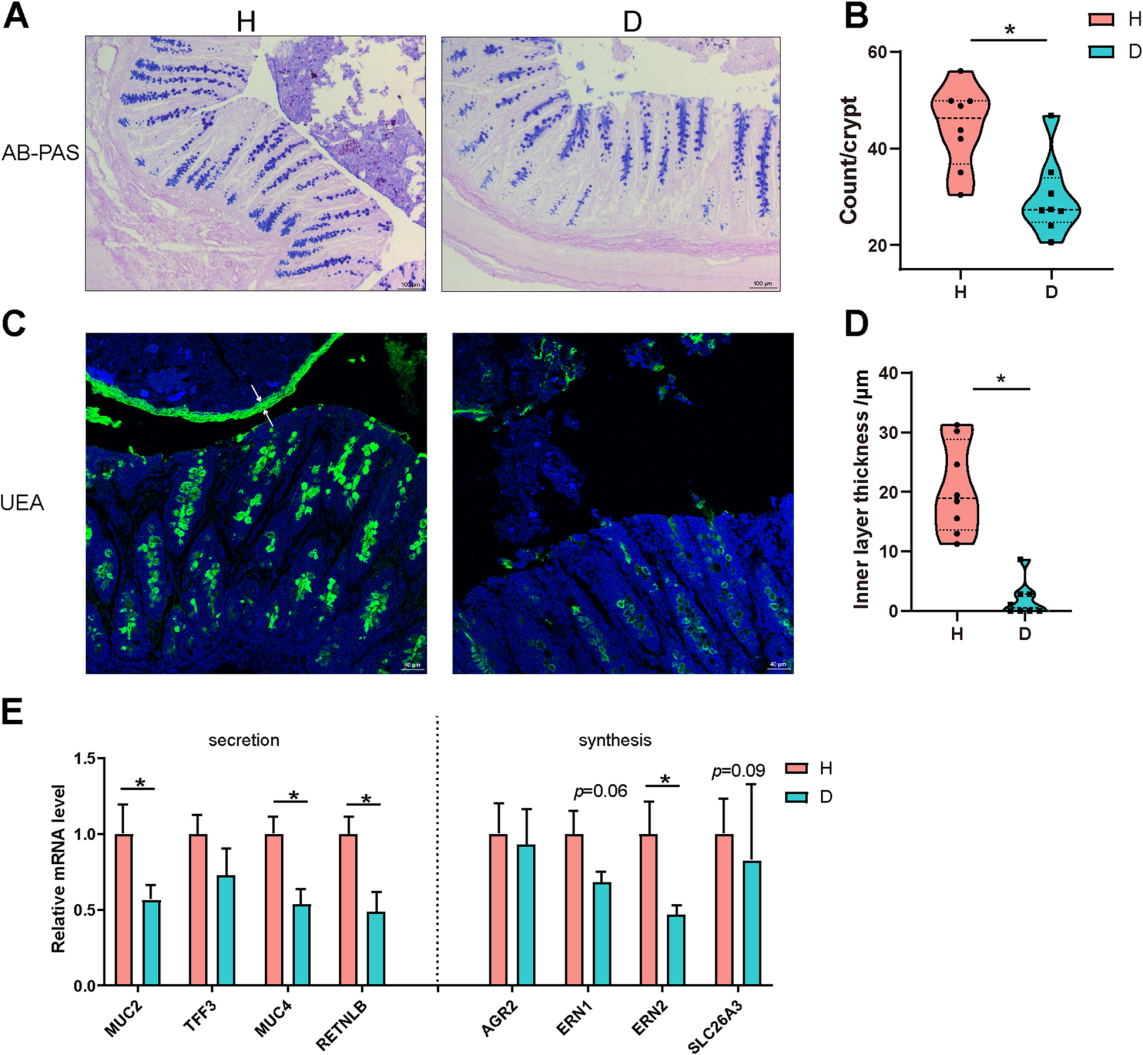
Diarrheal piglets exhibit a breached inner mucus layer. **A** Representative images of AB-PAS-stained colonic sections. Scale bar = 100 μm. **B** The number of goblet cells in the colon. **C** Representative UEA staining of colonic sections. White arrows point to the inner mucus layer. Scale bar = 40 μm. **D** The inner mucus layer thickness of colon. **E** The mRNA levels of mucus-related genes. Data are presented as mean ± SE or min to max showing all points. H, healthy controls; D, diarrheal piglets; AB-PAS, Alcian blue-periodic acid-Schiff; UEA, Ulex europaeus agglutinin

The intestinal mucus layer is the first line of host defense against both encroaching commensal bacterial and invading enteric pathogens [6]. As shown in Fig. 1A–D, the thickness of mucus layer and the number of goblet cells were greatly reduced in diarrheal piglets as shown by an Ulex Europaeus Agglutinin (UEA) immunofluorescence assay and Alcian blue-periodic acid-Schiff (AB-PAS) staining. Consistent with these results, diarrheal piglets showed a reduction in mucus-related genes, including *MUC2*, *MUC4*, *RETNLB*, *ERN1*, *ERN2*, and *SLC26A3*, in the colonic mucosa (Fig. 1E).

**Fig. 2:**
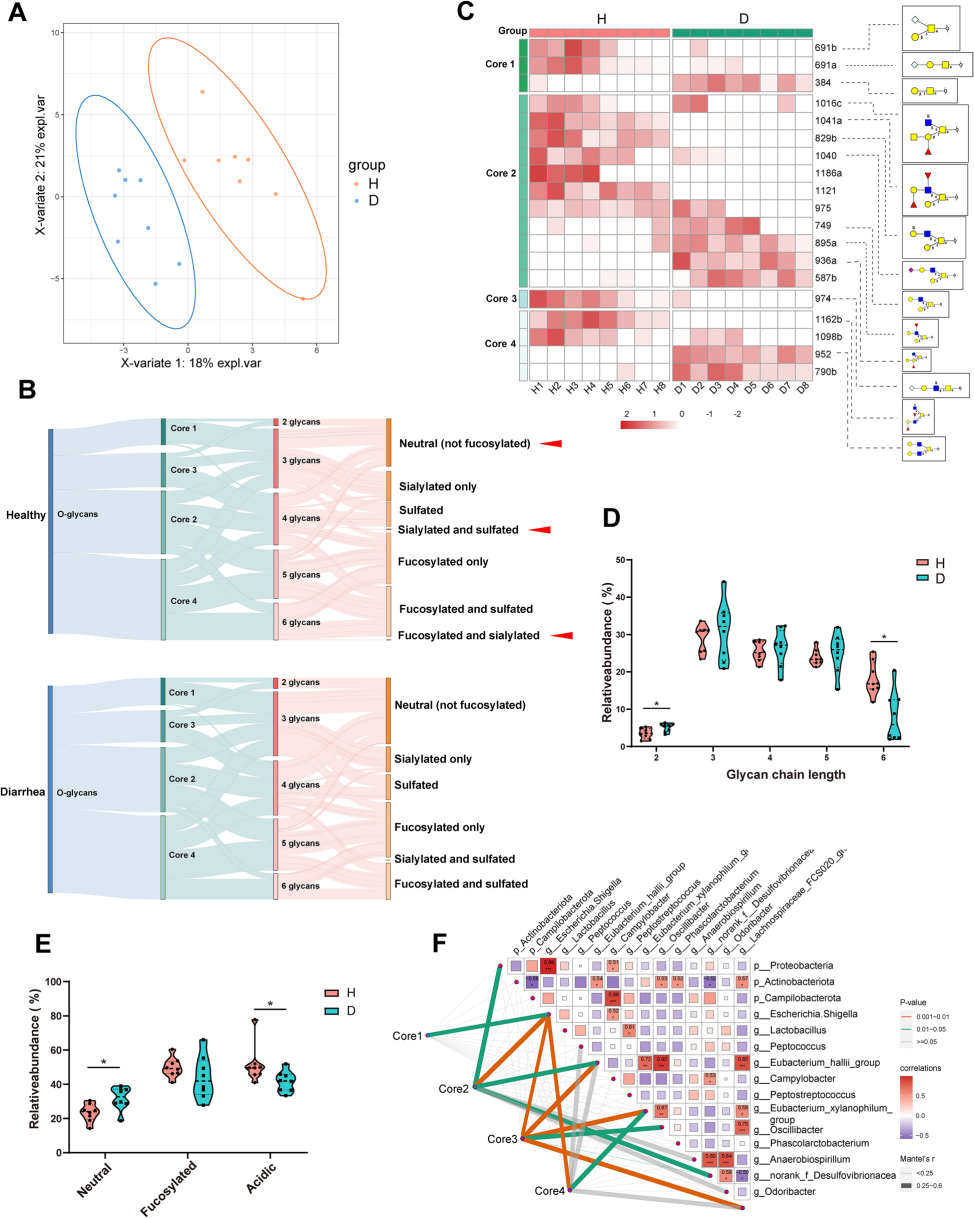
Diarrheal piglets show abnormal mucin *O*-glycan profile. **A** PLS-DA of mucin *O*-glycan profile in the colon. **B** Sankey flow diagram of *O*-glycan structures in two groups. The width of the flow corresponds to the relative abundance of structures. Red arrowhead indicates structures altered in piglets. *P*<0.05. **C** Heatmap illustrating the altered relative abundance of mucin *O*-glycans. *P*<0.1. Data are presented as relative abundance. The relative abundance of **D***O*-glycan chain length and **E***O*-glycan structures in the colon. Data are presented as min to max showing all points. **F** Pairwise comparisons of differently abundant taxa are shown, with a color-gradient denoting Spearman’s correlation coefficients. *O*-glycans are related to each taxa by partial Mantel tests. Edge width corresponds to the Mantel’s statistic for the corresponding distance correlations, and edge color denotes the statistical significance. H, healthy controls; D, diarrheal piglets; PLS-DA, partial lease-squares discriminant analysis

A glycomic analysis using PGC-LC-MS/MS was conducted to examine the overall mucin *O*-glycan compositions in the colon. A total of 57 unique *O*-glycan structures were identified by interpretation of the MS/MS fragmentation spectra (Table S2). Partial least squares discriminant analysis (PLS-DA) model exhibited a significant separation of clustering pattern between the two piglet groups (Fig. 2A). Among the mucin *O*-glycans from both diarrheal and healthy controls, core 1 and core 3 glycans were lower in abundance than core 2 and core 4 glycans (Fig. 2B). Core 2 and core 4 type glycans dominated the glycan spectra, with a higher amount of core 4 glycans and a larger number of core 2 branched glycoforms in the colon of piglets (Fig. 2B, Table S2). Out of the 57 *O*-glycan structures identified in this study, 19 glycans were affected by diarrhea in the colon (Fig. 2C). The relative abundance of core 1 glycans: 691a and 691b was significantly lower in the diarrheal piglets compared to the healthy controls, whereas the relative abundance of glycan 384 was substantially increased. Among core 2 glycans, the relative abundance of glycans 829b, 1016c, 1040, and 1041a was decreased in diarrheal piglets compared with their healthy counterparts, and the glycans 975, 1121, and 1186a also showed a similar trend. The relative abundance of glycans 749, 895a, and 936a were also elevated, and the glycan 587 exhibited a similar trend in diarrheal piglets. Only one core 3 glycan 974 was significantly lower in diarrheal piglets compared to the healthy controls. Of all core 4 glycans, two glycans were significantly different, and glycan 1162b was found to be less abundant in diarrheal piglets compared to the healthy control and glycan 952 was found to be more abundant. Although the relative abundance of core 4 glycans 1098b and 790b in diarrheal piglets followed a similar trend as glycans 1162ba and 952, the differences in abundance between the two groups did not reach statistical significance. In addition, core 2 glycan 1186a and core 4 glycan 1162b were only detected in the healthy piglets (Fig. 2C, Table S2).

Mucin *O*-glycans can also be broadly classified into neutral, fucosylated, and acidic glycans, which are categorized further into sulfated or sialic [37]. We observed an increased neutral glycans and a decreased abundance of acidic glycans in the colon of diarrheal piglets (Fig. 2E). Interestingly, *O*-glycan structures with both fucosylation and sialylation were only identified in the colon of the healthy controls (Fig. 2B). Consistently, a prominent decrease in the relative mRNA expression of fucosyltransferase *FUT1*, sialytransferase *ST6GAL1*, and sulfotransferase *GAL3ST1* in the colon of diarrheal pigs were observed (Fig. S5 A). The glycan chain length on mucin from both diarrheal and healthy piglets ranged from 2 to 6 monosaccharides (Fig. 2D). Relative amounts of short glycans (dissaccharides) were more abundant in the colon of diarrheal piglets while longer glycans (hexasaccharides) were less abundant (Fig. 2D). The mRNA expression of glycosyltransferases that function to elongate the glycan structures, including *GCNT2*, *B3GNT3*, *B3GALT5*, *B4GALT3*, *B4GALT6*, and *B4GALT7,* were found to be reduced in the colon of diarrheal piglets (Fig. S5 B), indicating that mucin *O*-glycans from diarrheal piglets would less likely be elongated to polysaccharides.

### Diarrhea leads to compromised colonic barrier function

**Fig. 3:**
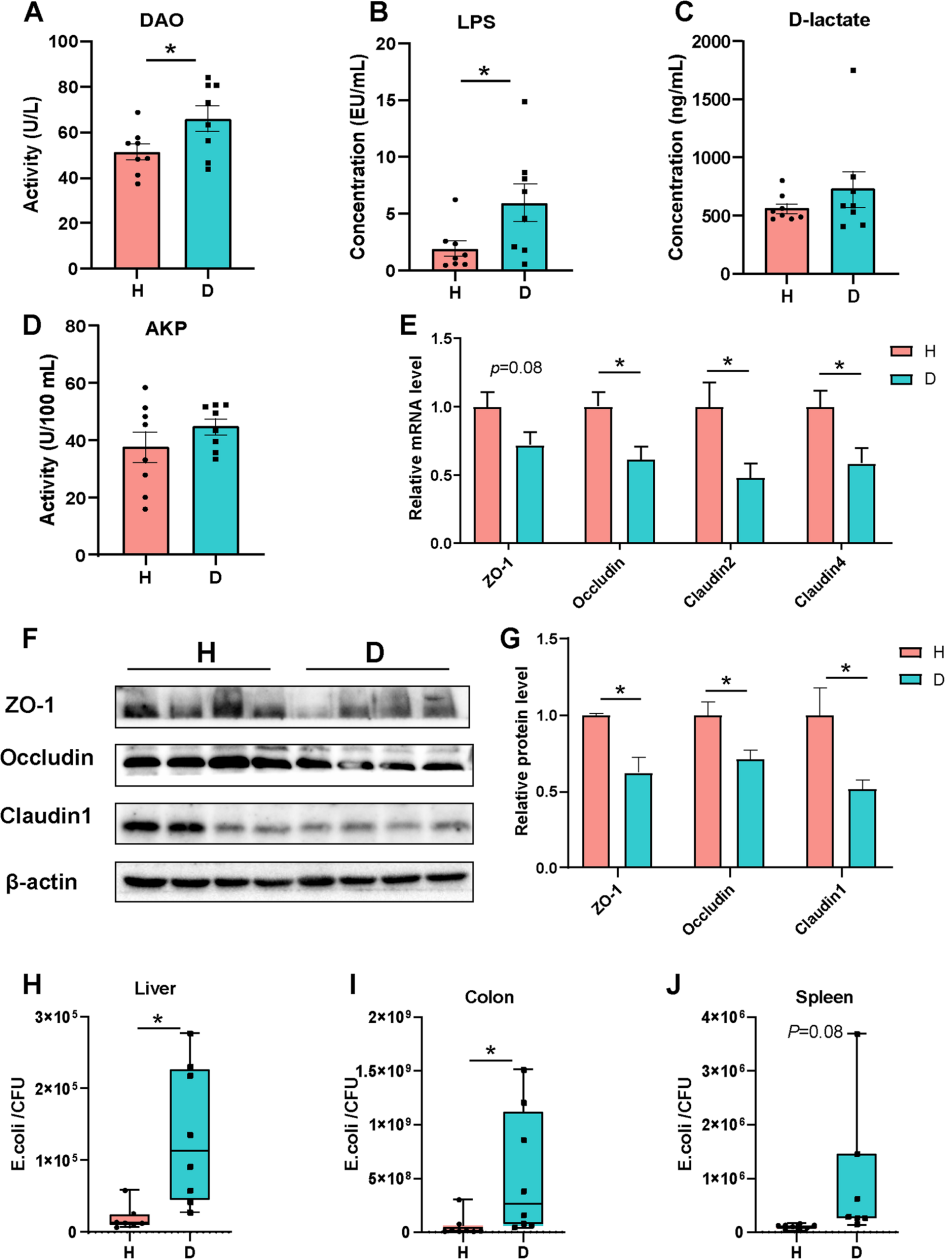
Diarrheal piglets show impaired colonic epithelial barrier function. The activities of **A** DAO and **D** AKP in serum. The concentrations of **B** LPS and **C** D-lactate in serum. **E** The mRNA levels of *ZO-1*, *Occludin*, *Claudin2*, and *Claudin4* in the colon. **F** Immunoblot analysis of tight junction proteins in the colon of piglets. **G** The protein levels of ZO-1, Occludin, and Claudin1 in the colon. qRT-PCR analysis of pathogenic *E. coli* detected in the **H** liver, **I** colon, and **J** spleen. Data are presented as mean ± SE or min to max showing all points. H, healthy controls; D, diarrheal piglets; DAO, diamine oxidase; AKP, alkaline phosphatase

**Fig. 4:**
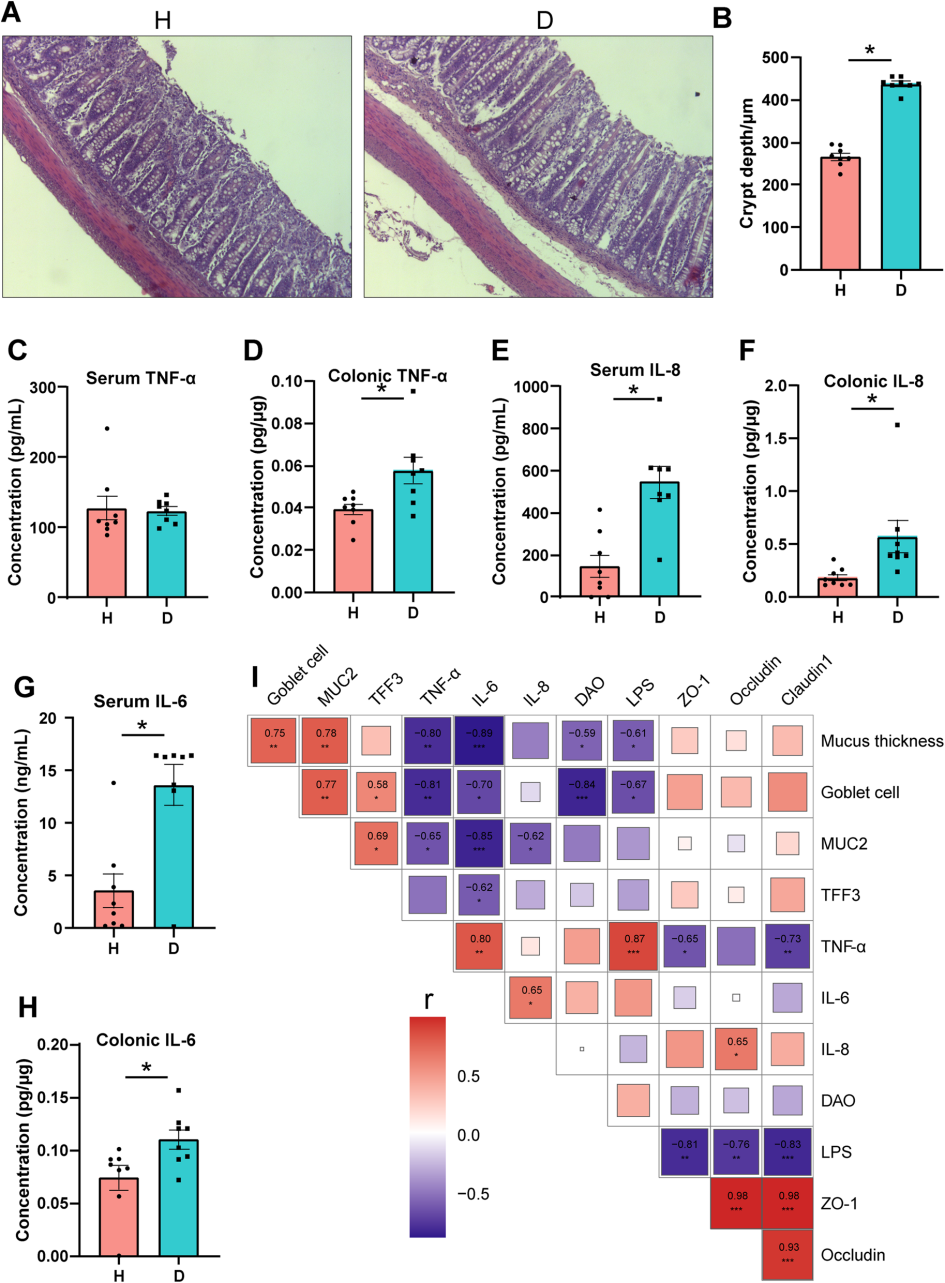
Systemic inflammatory response induced by diarrhea in piglets. **A** Representative H&E staining of colon of piglets. Scale bar = 100 μm. **B** Colonic crypt depth in the two groups. The levels of TNF-α in **C** serum and **D** colon. The levels of IL-8 in **E** serum and **F** colon. The levels of IL-6 in **G** serum and **H** colon. **I** Correlation coefficients between mucus layer, inflammatory response, and epithelial barrier. Correlation coefficients >0.5 or ≤0.5, *P<0.05, **P<0.01. Firebrick and navy colors denote positive and negative correlations, respectively. Color intensity is proportional to Spearman’s rank correlation values

Based on the aforementioned results, diarrhea is associated with mucus secretion defects and aberrant mucin *O*-glycans. As presented in Fig. 3A–G, diarrheal piglets showed a leaky gut with higher levels of serum diamine oxidase (DAO) and lipopolysaccharide (LPS), decreased abundance of tight junction proteins ZO-1, Occludin, and Claudin1 as well as a decrease in expression of genes encoding for *ZO-1*, *Occludin*, *Claudin2*, and *Claudin4*. Diarrhea also led to *E. coli* translocating into the liver, spleen, and colon tissue (Fig. 3H–J). No difference in the copy number of total bacteria was observed between the two groups (Fig. S6 E). H&E staining revealed that diarrheal piglets displayed notable crypt hyperplasia as indicated by the crypt depth (Fig. 4A–B). Moreover, increased severity of inflammation in diarrheal piglets was characterized by elevated levels of cytokines, including serum and colonic IL-6, serum and colonic IL-8, serum IL-1β, and colonic TNF-α (Fig. 4C–H, Fig. S6 A-D). The Data Integration Analysis for Biomarker discovery using a latent component method for Omics (DIABLO) revealed that *Eubacterium hallii group* was positively associated with the metabolite butyrate, Occludin, and ZO-1, but negatively associated with LPS. *Lactobacillus* was negatively correlated with Occludin and Claudin1 but positively linked to lactate, which was in turn positively correlated to LPS. *Escherichia Shigella* was negatively correlated with the ZO-1, Claudin1, LCA, and DCA were positively correlated with LPS that was in turn positively correlated to the phyla *Proteobacteria* (Fig. S6 F).

A correlation test by taking the mucus layer, cytokines, and tight junction proteins use into account was employed to investigate the correlation between the mucus layer and epithelial barrier function in piglets (Fig. 4I). We found that the abundance of tight junction proteins (ZO-1, Occludin, and Claudin1) were significantly correlated with the concentration of LPS. The thickness of inner mucus layer and the number of goblet cells were negatively correlated with increased severity of inflammation (IL-6 and TNF-α) and intestinal permeability (DAO and LPS).

### The mucin O-glycan-microbiota axis contributes to commensalism and gut homeostasis

Mantel’s correlation analysis showed that *Escherichia Shigella* had a robust relationship with mucin *O*-glycans, including core 1, core 2, core3, and core 4 structures. Core 2 and core 3 had a strong correlation with the phyla Proteobacteria, and the genera *Eubacterium hallii group* and *Eubacterium xylanophilum group* (Fig. 2F).

**Fig. 5:**
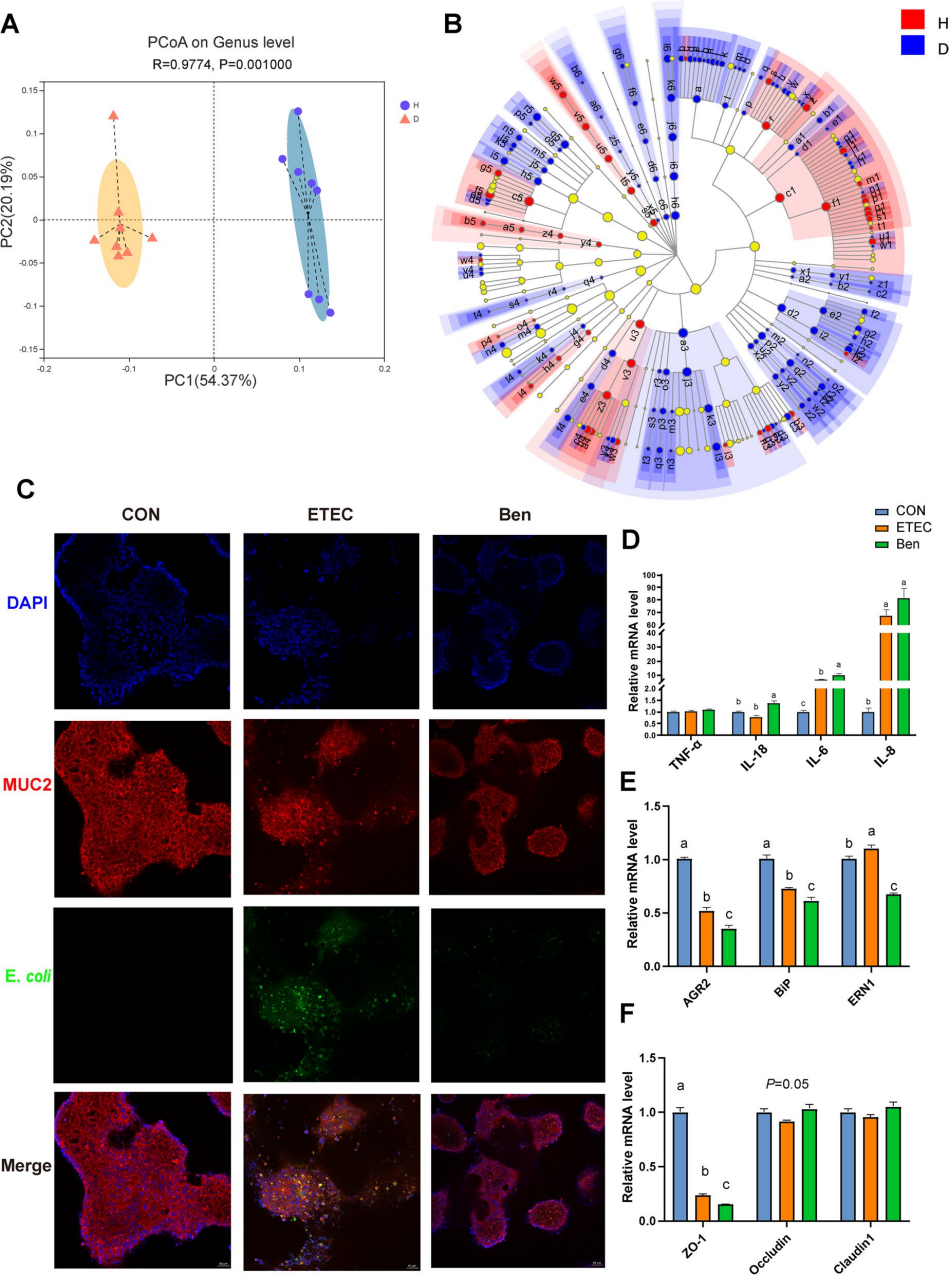
The mucin *O*-glycans-microbiota axis contributes to gut homeostasis. **A** Weighted UniFrac PCoA plots of colonic mucin fermentation in vitro. **B** Discriminating taxa between the mucins from diarrheal piglet and healthy control fermentation, as determined by LEfSe analysis (LDA≥2). **C** Representative images of *E. coli* k88 adherent to the T84 cells after treatment with mucin *O*-glycans inhibitor benzyl-α-GalNAc. The mRNA levels of **D** inflammatory cytokines, **E** ER-related genes, and **F** tight junction protein-related genes in the T84 cells after treatment with mucin *O*-glycans inhibitor benzyl-α-GalNAc. Data are presented as mean ± SE. H, healthy controls; D, diarrheal piglets; CON, control group; ETEC, *E. coli* k88 group; Ben, *E. coli* k88+Ben-α-Gal group

To seek relationship between mucin *O*-glycan and gut microbiota, we examined the alteration of SCFAs and microbiome after the incubation of fresh piglet feces with colonic mucins from diarrheal piglets or their healthy counterparts using an in vitro fermentation system. Fermentation of colonic mucin glycans (CMGs) from diarrheal piglets by the gut microbiota resulted in a remarkable reduction of acetate, propionate, isobutyrate, butyrate, isovalerate, and valerate (Fig. S7 A-F). A 16S rRNA sequence analysis showed that CMGs from diarrheal piglet fermentation harbored a different microbiome in both α- and β-diversity within bacterial communities as compared to the healthy controls (Fig. 5A, Fig. S7 G-J). Broad population changes were found, ranging from the phylum to genus levels, in comparisons of CMGs from diarrheal piglets and their healthy controls fermentation (Fig. 5B, Table S3). Analyses of the microbiota at the phylum level revealed a significant increase in the relative abundance of *Desulfobacterota* and a decrease in the relative abundance of *Fusobacteriota* in the diarrheal piglets’ CMG fermentation compared to the healthy controls. Taxonomic classifications at the genus level revealed that CMGs from diarrheal piglets exhibited highly enriched *Desulfovibrio* and *Lactobacillus*, whereas *Prevotella*, *Ruminococcus torques group*, and *Faecalibacterium* were reduced after 24h fermentation. Notably, at the genus level, the abundance of *Blautia* and *Subdoligranulum* were also significantly decreased in CMGs from diarrheal piglet fermentation, whereas the abundance of *Parabacteroides*, *Phascolarctobacterium*, and *Bacteroides* increased.

To directly assess the potential roles of mucin *O*-glycan in pathogenic adhesion, benzyl-α-GalNAc was employed to inhibit mucin *O*-glycosylation. As expected, the fluorescence staining indicated a significant reduction in the number of *E. coli* k88 adherent to the mucin-producing cell line T84 cells after 72h treatment with the inhibitor (Fig. 5C), substantiating mucin *O*-glycans acting as anchor sites for bacteria [7]. We also noticed that key genes (*ARG2*, *BiP*, and *ERN1*) related to endoplasmic reticulum-resident proteins showed a decrease in expression after being incubated with benzyl-α-GalNAc (Fig. 5E). The disrupted epithelial barrier function and inflammation susceptibility were further illustrated and supported by the induced cytokines, including expression of *IL-18*, *IL-6*, *IL-8*, and repressed *ZO-1* expression (Fig. 5D, F).

### Diarrhea is associated with inflammasome activation and autophagy restriction

**Fig. 6:**
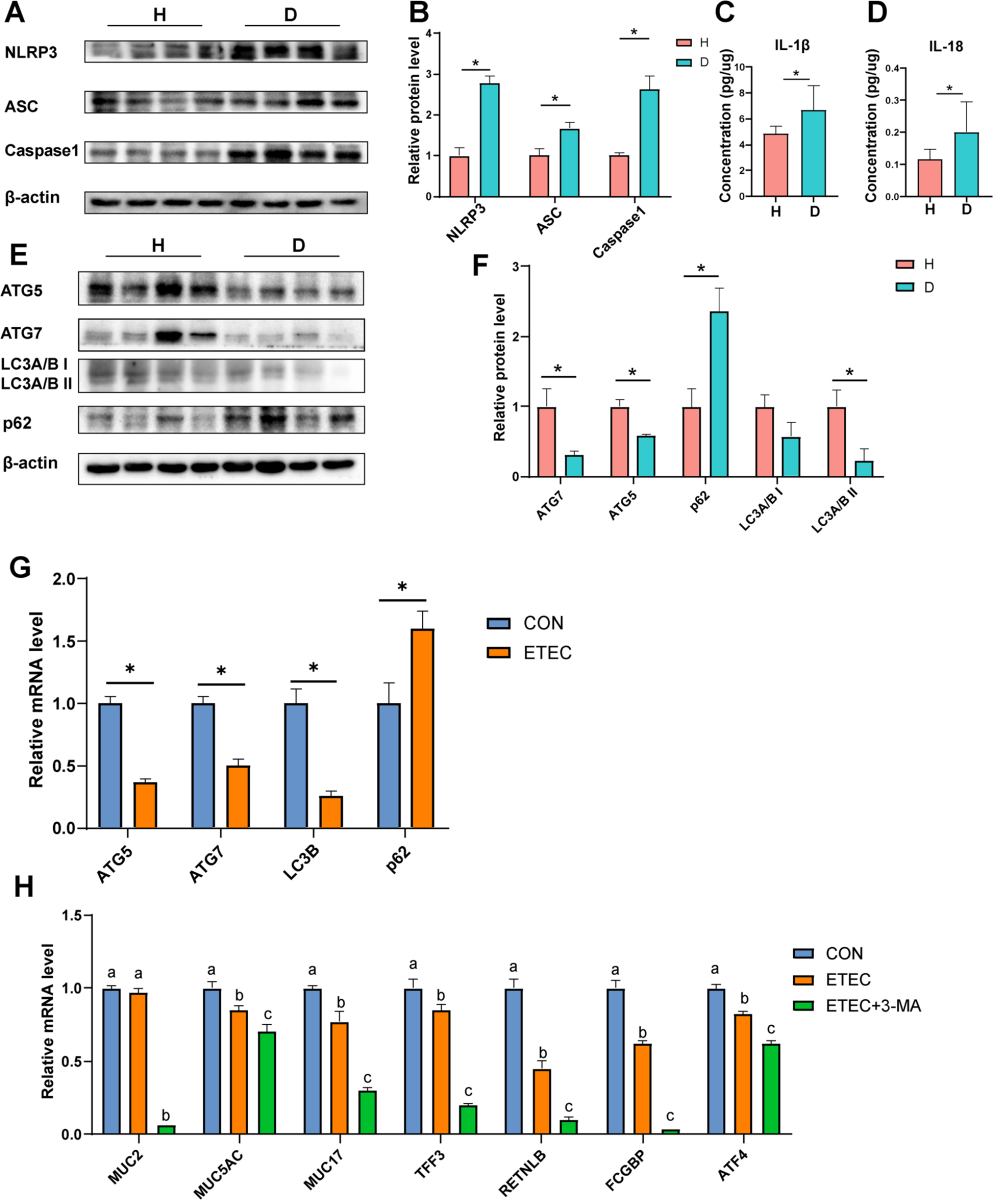
The mechanisms underlying impaired mucin *O*-glycans in diarrheal piglets. **A** Immunoblot analysis of the colon of piglets. **B** The protein levels of NLRP3, ASC, and Caspase 1 in the colon. The concentrations of **C** IL-1β and **D** IL-18 in the colonic tissues. **E** Immunoblot analysis for the colon of piglets. **F** The protein levels of ATG5, ATG7, LC3A/B, and p62 in the colon. **G** The mRNA levels of *ATG5*, *ATG7*, *LC3B*, and *p62* in the T84 cells after infection with *E. coli* k88. **H** The mRNA levels of *MUC2*, *MUC5AC*, *MUC17*, *TFF3*, *RETNLB*, *FCGBP*, and *ATF4* in the T84 cells after treatment with 3-MA. Data are presented as mean ± SE. H, healthy controls; D, diarrheal piglets; 3-MA, 3-methyladenine; CON, control group; ETEC, *E. coli* k88 group; ETEC+3-MA, *E. coli* k88+3-MA

Having shown that mucin *O*-glycan and microbiota reciprocally regulated each other, we explored the potential downstream mechanisms related to the impaired mucin *O*-glycosylation in diarrheal piglets. It has been reported that the inflammasome complex and autophagy affect mucus secretion by goblet cells and thus the intestinal barrier [4]. We observed the elevated expression of inflammasomes, including NLRP3, ASC, and Caspase 1, in diarrheal piglets (Fig. 6A, B). The inflammasome complex recruits the adaptor protein ASC, thereby activating Caspase 1 and triggering the release of mature IL-1β and IL-18 [38]. Consistent with the inflammasome activation, analysis of colonic tissues revealed increased IL-1β and IL-18 released from diarrheal piglets compared to the healthy controls (Fig. 6C, D). Moreover, autophagy, downstream of inflammasome signaling, is critical for goblet cell secretory function. Western blotting analyses revealed that piglets with active diarrhea reduced levels of ATG7, ATG5, and LC3A/B II and accumulated p62 in the colon, indicative of diminished autophagosome formation (Fig. 6E, F).

Given the fact that diarrhea affected autophagosome formation in piglets, the effects of *E. coli* k88 challenge on T84 cells were assessed. Compared with the control (CON) group, a higher mRNA level of p62 was detected in the *E. coli* k88 (ETEC) group, whereas lower mRNA levels of *ATG5*, *ATG7*, and *LC3B* were detected (Fig. 6G). Moreover, pretreatment with the autophagy inhibitor 3-methyladenine (3-MA) suppressed the expression of key genes (e.g., *MUC2*, *TFF3*, *MUC17*, and *RETNLB*) related to goblet cell secretions (Fig. 6H).

## Discussion

The secretion of mucins by goblet cells establishes a mucus layer that segregates the microbiota from the intestinal epithelium and is predominantly made up of the highly glycosylated mucins [5]. *O*-glycans, constituting up to 80% of the mucin by mass weight, are especially critical in regulating the gut microbiota by providing bacterial ligands and nutrients and ultimately maintaining intestinal barrier integrity [39]. While it is widely recognized that diarrhea is associated with the gut microbiota dysbiosis and growth deficit, the role of mucin *O*-glycans in regulating gut homeostasis has not been investigated. One of the hurdles causing this knowledge gap in the field is the realistic challenge to conduct experiments in human infants due to ethical constraints. Early weaned piglets are prone to develop diarrhea. The porcine intestinal epithelium, immune system, and gut microbiota at birth development stage are more mimic to human infants than neonatal mouse pups [40, 41]. In order to fill this knowledge gap, we have taken advantages of a piglet model to investigate the mucin *O*-glycans changes associated with gut microbial community and their mechanisms linked to some pathophysiological features of diarrhea.

The function of intestinal barrier relies on crosstalk among the three components: the commensal microbiota, the mucus layer, and the intestinal epithelium [1]. The latter two components affect symbiotic bacterial colonization, and their impairment might trigger inflammation. The inner colonic mucus layer is well defined and attached to the epithelial surface, thus preventing microbes from reaching and translocating in the intestinal epithelium [42]. However, as shown in our study, the inner layer was fully deteriorated in the diarrheal piglet group. Mucus layer degradation was also observed in mice infected with gut pathobiont *Citrobacter rodentium* [11] and in patients with active ulcerative colitis [43]. Previous studies have found that supplementation with *Bifidobacteria* or inulin could prevent colonic mucus defects [44, 45], indicating that it may be possible to design probiotics or prebiotics for potential treatment of mucus layer deterioration in diarrheal children. The intestinal epithelial barrier function is related to many factors, including D-lactate, endotoxins in serum, and tight junction proteins [46]. Our results showed that diarrhea led to enhanced intestinal permeability and poor epithelial barrier function, as evidenced by higher concentrations of DAO and LPS in the serum and lower expression of ZO-1, Occludin, Claudin1, Claudin2, and Claudin4 in the colon, mirroring the previous findings [47]. PCoA also showed a distinct clustering pattern between the samples from diarrheal piglets and healthy controls. The dysbiosis of colonic microbiota was associated with the reduction in commensal bacteria and increase in opportunistic pathogenic microorganisms. Mounting pieces of evidence have endorsed the concept that a bloom of *Proteobacteria* in the gut reflects dysbiosis or an unstable microbial community structure, which usually can be found in mice with an increased inflammation [14, 48, 49]. Notably, we reported an increased abundance of several well-known pathogens such as *Escherichia Shigella*, *Peptococcus*, and *Campylobacter* in the diarrheal group, which is in line with previous studies of children and rhesus macaque with diarrhea [50, 51] as well as an immunosuppressive mouse model [52]. We did observe the appearance of the gut pathobiont, *E. coli*, in the liver, colonic mucosa, and spleen in piglets with active diarrhea, suggesting that *E. coli* is a leading cause of post-weaning diarrhea [53].

Bacteria-derived metabolites of SCFAs are known to maintain intestinal barrier integrity and protect from intestinal inflammation. Butyrate, in particular, has been reported to restore the delocalization of tight junction proteins Occludin and F-actin [14] and enhance the expression of tight junction proteins by activating adenosine monophosphate-activated protein kinase (AMPK) activity [54]. SCFAs also mediate immunosuppression by either enhancing mTOR and STAT3 signaling-mediated anti-microbial factors [55]. The abolished butyrate-producing bacteria *Eubacterium hallii group* may further cause the deterioration of the intestinal barrier in the diarrheal piglets. BAs are essential physiologic molecules, which are synthesized in the liver, reserved in the gallbladder, and released into the intestine [56]. Several species of gut bacteria, including *Clostridium*, *Lactobacillus*, *Bifidobacterium*, and *Bacteroides* are capable of converting PBAs CA and chenodeoxycholic acid (CDCA) into SBAs DCA and LCA [57]. Studies analyzing the fecal BA profile revealed that total levels of BAs were elevated in irritable bowel syndrome-diarrhea (IBS-D) when compared with the healthy controls. Moreover, an increased proportion of PBAs, especially CDCA, in IBS-D, and decreased levels of SBAs including DCA in irritable bowel syndrome-constipation (IBS-C) were observed [58, 59]. Altered BA metabolism impacts intestinal inflammation, as demonstrated in IBD patients, with decreases in Firmicutes and increases in *Lactobacillus* and *Enterobacteria* (*E. coli* at specie level) during flares [24]. A previous study found higher concentrations of PBA CDCA in the stool of patients with bile acid diarrhea and further showed that immune activation or inflammation and increased permeability could conceivably be contributing to the onset of diarrhea [60]. Our study showed a higher PBA CA concentration and decreased levels of SBAs LCA and HDCA in piglets with diarrhea compared to in the healthy controls. These results combined with the elevated proinflammatory cytokines, including IL-6, IL-8, and TNF-α support the notion that diarrhea causes colonic microbiota dysbiosis, bacterial translocation, and subsequent leakage into other visceral organs, ultimately leading to colonic barrier dysfunction. Intriguingly, there is a disproportionate amount of the well-known probiotic *Lactobacillus* in diarrheal piglets, which is also observed in irritable bowel syndrome patients [61] and *Clostridium difficile*-associated diarrhea patients [62]. Lactate is primarily produced by lactic acid bacteria in the gut, such as *Lactobacillus* [63]. The correlation analysis in our study confirmed the trophic interaction between *Lactobacillus* and lactate production in the colon. The increased abundance of *Lactobacillus* and the accumulation of lactate in the colon may make diarrhea worse because lactate, as a natural organic acid, is very slowly absorbed by epithelial cells [64] and its cumulation leads to an osmotic load for water secretion from the mucosa in the large intestine [65].

As discussed above, the loss of the mucus layer results in bacterial translocation and disrupts intestinal homeostasis. The colonic mucus layer is heavily comprised of *O*-glycosylated mucins that are produced by goblet cells. All *O*-glycans are initiated with the attachment of GalNAc residues to the hydroxyl group of Ser and Thr residues on the protein backbone, which are then catalyzed by a polypeptide GalNAc-transferase (GALNT) [6]. After the initial addition of GalNAc, biosynthesis of core *O*-glycan structures occurs as a stepwise addition of Gal, GlcNAc, and GalNAc by glycosyltransferases, yielding higher order glycans [39]. In the colon of diarrheal piglets, shorter glycans, predominantly disaccharides, were highly abundant as the core 1 glycan 384, while longer glycans with hexasaccharides were less abundant, which was accompanied by lower levels of enzymes responsible for elongation of the core structures. The increased levels of a subset of smaller glycans in the patients with active ulcerative colitis [34] and pigs infected with *Brachyspira hyodysenteriae* [37] are related to both the inflammation and the severity of disease. Moreover, compared with conventionally raised mice, decreased glycosylation with less glycosyltransferases and truncated glycans were detected in the germ-free mice [66], indicating the role of the mucin glycans as possible interaction partners for bacteria. We found that the levels of SCFAs were significantly lower after the incubation of CMGs with feces from diarrheal piglets in an in vitro fermentation assay, confirming that normal mucin *O*-glycans were utilized as an endogenous fermentation source to produce SCFAs [67]. It has previously been reported that mucin *O*-glycans enhance SCFAs production, but also promote mucosal immune homeostasis [68]. A previous study also demonstrated that exogenously provided mucin *O*-glycans mitigated perturbations to the microbiota including suppressing *Clostridium difficile* and increasing *Akkermansia muciniphila* abundance and reducing the overconsumption of the host mucus [69]. These findings support the notion that exogenous mucin *O*-glycans provide benefits to host physiology and help prevent intestinal mucus from being compromised in infants with diarrhea. The normal CMG fermentation also increased the proportions of *Faecalibacterium*, *Prevotella*, and *Blautia*, which have been reported to be associated with producing SCFAs [70]. The higher levels of the phyla *Desulfobacterota*, the class *Desulfovibrionia*, and the genera *Desulfovibrio* were observed in the CMGs from the diarrheal piglet fermentation group in our study, which are associated with a penetrable mucus phenotype in animals with a higher inflammatory disease [71]. We also found relatively higher proportions of the genera *Bacteroides* and *Parabacteroiodes* in the aberrant CMG fermentation group, bacteria that have been found to be increased in celiac disease patients [72] and mice with less developed mucus layer [71]. Consistent with the in vivo results, we observed that aberrant CMG fermentation also resulted in a significant increase in the relative abundance of *Lactobacillus*, further highlighting the connection with less developed mucus layer, bacteria, and diarrheal phenotype.

The mucin *O*-glycan structures are commonly terminated by sulfate, fucose, and sialic acid residues. The terminal display of sialic acid and sulfate sequester pathogens act as binding targets and modulate adhesion. It has been reported that sialic acid residues on mucin can offer a favorable environment and provide a source of nutrients for certain microbes [8]. Terminal residues such as sulfate and Neu5Ac are essential in the enhanced mucus viscosity and structural integrity of the mucus layer [73], as the removal of terminal Neu5Ac is an initial step in the sequential degradation of mucin glycans [74]. Certain bacteria, known as mucin-degrading bacteria, are capable of digesting glycans by secreting glycosidase enzymes such as sialidases, glycosulfatases, fucosidases, endo-β-*N*-acetylglucosaminidases, and β-galactosidases. It has been reported that sialidases play a key role in the ability of *Ruminococcus torques* and *Fusobacterium* to utilize mucins as a nutrient source [75]. A comparative genomic analysis revealed that *Ruminococcus torques* enabled to cleave 56 glycan structures and utilized MUC2 as a sole carbon source [76]. Continual glycan degradation mediated by glycosidases may lead to the disappearance of host-specific glycan epitopes [77] and increased host susceptibility to pathogens that lack the mucin-degrading capability. The expansion of pathogens during diarrhea such as *E. coli* may rely on sialic acid released from mucin glycans catalyzed by sialidases in mucin-degrading bacteria. Our results from the in vitro fermentation demonstrated that reduced terminal sialic acid and sulfate residues paralleled an outgrowth of the increased various pathogens and reduction in the proportions of *Ruminococcus torques* and *Fusobacterium* due to loss of the energy source, further confirming a mutualistic relationship between the host glycans and gut microbiota. Mucin sulfation has also been proposed to offer additional protection from luminal insults by increasing mucus viscosity and resistance to bacterial degradation. The physiological significance of mucin sulfation was addressed by increased infiltration of CD45^+^ inflammatory cells and necrotic lesions as well as hemorrhagic diarrhea in the GlcNaC6ST-2-deficient mice administrated by dextran sulfate sodium (DSS) [78] and pigs infected with *Brachyspira hyodysenteriae* [37]. Another study used mice deficient in the sulfate transporter NaS1, and found that the mice suffered from an impaired intestinal barrier function and elevated disease activity [79]. We found that *O*-glycans being terminally sialylated and sulfated were found in lower amounts in the colon of diarrheal piglets, which partially explains defects in the mucus layer and intestinal barrier. Additionally, *O*-glycans terminating with fucose and sialic acid were found in significantly less abundant in the colon of diarrheal piglets compared to that of the healthy controls. Fucose epitopes are a protective mechanism that protects the host from pathogenic infection by downregulating virulence gene expression of pathogens and inhibiting adherent [80], this suggests that the decrease in fucose and sialic acid may be another hallmark of gut microbiota dysbiosis.

We next sought to dissect the potential mechanisms of the mucus secretory defects and aberrant mucin *O*-glycans as related to diarrhea. The inflammasome is a multi-protein complex that is expressed in various cell lineages and orchestrates diverse functions in homeostasis and in response to inflammation. Inflammasome complexes, such as NLRP3, NLRC4, and NLRP6, recruit the adaptor protein ASC and activate Caspase 1, which subsequently cleaves and releases IL-1β and IL-18 [38, 81]. It has been reported that NLPR6 inflammasome influences intestinal barrier function and microbial homeostasis by regulating mucus secretion. Mice that were deficient in NLRP6, ASC, or Caspase 1 lacked a continuous mucus layer, resulting in an increased susceptibility to enteric infection [4]. Strikingly, we found a significant increase in the abundance of NLRP3, ASC, and Caspase 1 in the colon of diarrheal piglets compared to that in the healthy controls, which trigger the release of proinflammatory cytokines IL-1β and IL-18. Although NLRP3 inflammasome is one of the most extensively studied inflammasomes, the regulatory mechanisms of NLRP3 inflammasome activation are unclear [82]. Our results support the notion that the NLRP3 inflammasome was activated by some potential pathogen-derived stimuli in diarrheal cases in which the abnormal mucin *O*-glycan profile, breached inner mucus layer, and colonic barrier dysfunction were concurrently observed. Thus, it is conceivable that NLRP3 inflammasome appears to regulate mucus secretion affecting intestinal barrier function and microbial homeostasis. The activated NLRP3 inflammasome can help resist the damage of some pathogens. If, however, it is overactivated, it will trigger destructive inflammation. These findings in our study could shed light on the development of specific inhibitors to target inflammasome candidate as a novel strategy for potential therapy of diarrheal and intestinal diseases. Furthermore, autophagy has been identified as a main regulator of inflammasomes [4]. Many previous studies have shown that autophagy can inhibit the NLRP3 inflammasome activation by removing endogenous inflammasome activators [83]. In particular, some of the key autophagy proteins ATG3, ATG5-ATG12, and ATG7 are involved in converting the cytosolic protein LC3 I into phosphatidylethanolamine-conjugated LC3 II on the phagophore surface, a critical step in the formation of autophagosomes [84]. Deficiency in ATG5, ATG7, or LC3 led to mucin accumulation in goblet cells, resulting in mucus secretion defects [3]. In our piglet diarrhea model, we found a decreased abundance of ATG5, ATG7, and LC3A/B II, along with an increased accumulation of p62 in the colon that is a well-known substrate of autophagy [4]. *E. coli* k88 infection also inhibited autophagy by decreasing the expression ATG5, ATG7, and LC3B and increasing the expression of p62. These findings suggest dysfunction or dysregulated autophagy might occur and thus promote an inflammatory response in diarrheal pathogenesis. Furthermore, we found a reduction in goblet cell-specific transcript levels (e.g., *MUC2*, *TFF3*, *RETNLB*) that are main components of mucus layer in cells when treated with 3-MA blocking the autophagy pathway. This finding provided an additional piece of evidence that autophagy functions in regulating mucus secretion and therefore intestinal barrier function. It should be pointed out that the observed reduction in goblet cell-specific genes in our study is inconsistent with previous reports of no significant difference in gene expression in mice [4, 14]. This difference might reflect the discrepancy between mammalian in our study and rodent models. Overall, these combined findings collectively demonstrated that abnormal mucus secretion and *O*-glycans mediated by the autophagy-inflammasome pathway might be the reasons for colonic microbiota dysbiosis and barrier dysfunction.

## Conclusion

**Fig. 7:**
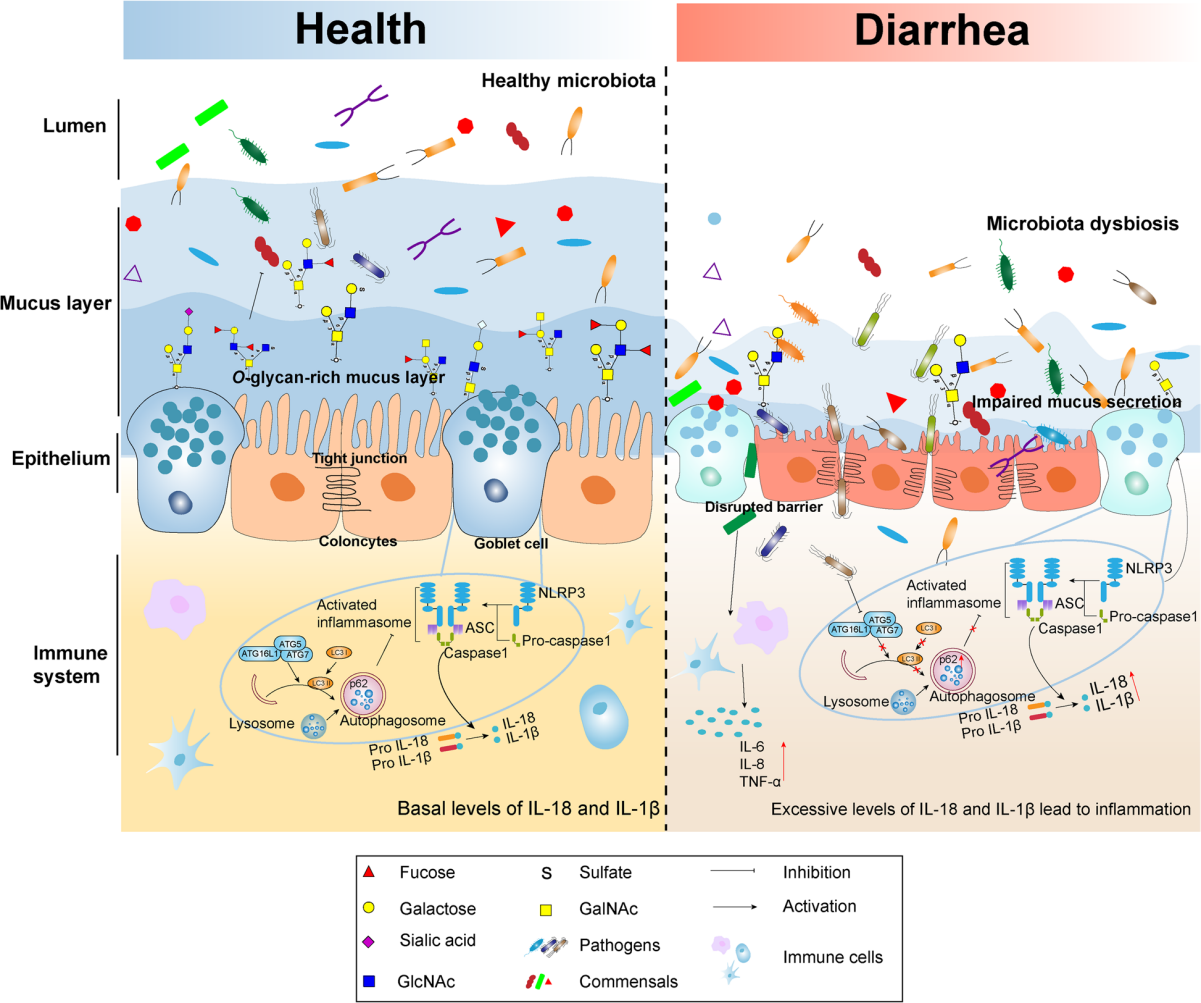
Mucin *O*-glycans-microbiota axis orchestrate gut homeostasis in healthy piglets (health). In the diarrheal piglet model (diarrhea), aberrant mucin *O*-glycans induced by inflammasome activation and autophagy restriction compromise gut microbiota dysbiosis, leading to epithelial barrier dysfunction

Our study using a piglet model with early-weaning-induced diarrhea provides evidence for the association of diarrheal disease with colonic microbiome and mucin *O*-glycan profiles that would not be possible in human infants. Taken together, we observed that diarrheal piglets exhibit colonic microbiota dysbiosis and mucosal barrier dysfunction. Our data demonstrated that diarrhea resulted in the activation of inflammasomes and autophagy defects along with aberrant mucin *O*-glycan structures including reduced acidic glycans and truncated glycans (Fig. 7). Maintenance of appropriate autophagy functions and the development of specific inhibitors to inflammasome may prevent *O*-glycans defects. These findings offer a new perspective on the pathogenesis and pathophysiology of diarrheal disease and suggest that preventing mucin *O*-glycans from defects and maintaining symbiotic interaction between glycans and gut microbiota may be an alternative strategy for the treatment of diarrhea and intestinal diseases in children.

## Materials and methods

### Animals and sample collection

All animal studies were approved by the Animal Welfare Committee in the Institute of Animal Science, Chinese Academy of Agricultural Sciences (Ethics Code Permit IAS 2020-104).

Six litters of neonatal piglets (Yorkshire) was chosen from a commercial farm (Henan, China) and weaned from their sow at 21 days of age. Six litters was raised in 6 individual houses that were equipped same mechanical ventilation, feed hopper, and slatted floors. All piglets had free access to food and drinking water. We observed the feces of the piglets daily and scored them according to the standard of fecal rating (Table S4). The feces of piglets with a score of 6 over 5 days were identified as active diarrhea. At 28 days of age, 8 piglets with active diarrhea were selected and gender-matched littermates were used for the 8 healthy controls (Table S5).

Serum samples were collected by centrifuging venous blood at 2000 × *g* for 15min, and the supernatants were stored at −80°C. After being euthanized with an injection of sodium pentobarbital (50 mg/kg BW) and exsanguination, the liver, the spleen, and the intestine were separated and weighed. A segment of the proximal colon measuring 1 cm was collected for histological analysis. After collecting the luminal digesta, the mucosa samples were collected by scrapings the intestinal wall with glass slides, frozen in liquid nitrogen, and stored at −80°C for subsequent mRNA and protein measurements.

### Cell culture and treatment

The human colon-derived T84 cell line was obtained from the ATCC (CCL-248). Cells were regularly maintained in Dulbecco’s modified Eagle’s medium F-12 (DMEM F-12) supplemented with 10% fetal bovine serum at 37°C, 5% CO_2_. *Escherichia coli* strains k88 was cultured in Luria-Bertani (LB) medium at 37°C.

T84 cells were seeded in 24-well plates and treated with the mucin *O*-glycan inhibitor benzyl-α-GalNAc (Sigma, St. Louis, MO) from stock in dimethyl sulfoxide (DMSO) to a final concentration of 2 or 10 mM autophagy inhibitor 3-methyladenine (3-MA) (Sigma, St. Louis, MO). After being challenged with *E. coli* k88 for 2h at a MOI of 100:1, the cells were collected using TRIzol reagent for mRNA expression measurements. For the bacterial adhesion assay, T84 cells were seeded onto 24-well culture plates containing poly-l-lysine-coated coverslips and treated with 2 mM benzyl-α-GalNAc for 72h or 10 mM 3-MA for 24h. After maintained in the LB broth at 37°C for 8h, *E. coli* k88 was fluorescently tagged with 100 μg/mL FITC in PBS at 37°C for 1h. The cells were pelleted by centrifugation at 10,000 g for 15min. *E. coli* k88 was washed in sterile PBS and centrifuged at 10,000 g for 15min three times. Finally, *E. coli* k88 was resuspended in DMEM F-12 supplemented with 10% fetal bovine serum incubated with T84 cells at 37°C for 2h. Coverslips were then washed with PBS three times and fixed in Carnoy’s fixative for 1h at room temperature. Cells were incubated with MUC2 antibody (Abcam, #ab90007) and then incubated with goat Cy3-conjugated anti-rabbit IgG (Solarbio, #K0034G) and followed by counterstained with DAPI (H-1200, Vector laboratories). Slides were viewed using confocal laser scanning microscopy (Leica TCS SP8, Germany).

### In vitro anaerobic culturing

Fresh feces from additional 8 piglets at 28 days of age were collected and pooled. Briefly, the piglets were selected from the same litter as the source of fecal inoculum. The feces were collected directly into plastic bags saturated with CO_2_. An equal amount of feces was taken from each piglet and mixed. The pooled feces were resuspended in sterile PBS and cocultured with mucin *O*-glycans from diarrheal piglets or their healthy counterparts under anaerobic conditions. The isolation of colonic mucin glycans was listed in glycomic analysis. After incubation for 24h, cultured samples were collected and used for SCFA detection and bacterial composition analysis.

### Biochemical analysis

D-lactate concentration and the activities of DAO and AKP in the serum were measured with commercial assay kits according to the manufacturer’s instructions (Nanjing Jiancheng Bioengineering Institute, Nanjing, China). Serum LPS concentration was detected using the commercial assay kit (Xiamen Bioendo Technology Co., Xiamen, China). The levels of IL-6, IL-8, IL-10, TNF-α, and IFN-γ in serum and colon tissue were measured using a specific enzyme-immunoassay technique (ELISA) following the manufacturer’s protocol (Thermo Fisher Scientific, Vienna, Austria). IL-1β and IL-18 concentrations were determined by an ELISA kit following the manufacturer’s protocol (Cusabio, Wuhan, China).

### RNA isolation and quantitative real-time PCR (qRT-PCR)

Colonic mucosa was homogenized by a homogenizer, and the total RNA was extracted with RNeasy Mini Kit according the manufacturer’s protocol (Qiagen, Hilden, Germany). cDNA was synthesized from 1 μg total RNA using the PrimeScriptRT reagent Kit (PrimeScript^TM^RT reagent Kit with gDNA Eraser, Takara). qRT-PCR was performed using the SYBR Premix Ex Taq II (Takara) on an ABI 7500 RT-PCR system (Applied Biosystems, Foster City, CA). All primer sequences are listed in Table S6. The mRNA levels were calculated using the 2^-ΔΔCT^ method and normalized to reference house-keeping genes *GAPDH* and *β*-*actin*.

### Histological analysis

For morphology, colonic tissues were freshly harvested from piglets and fixed with 10% neutral buffered formalin prior to paraffin embedding. Consecutive sections at 5-μm thickness were counterstained with hematoxylin and eosin (H&E). Morphological changes were examined under a DM300 light microscope (Leica, Germany). For analysis, ten crypts were randomly selected from different parts of the section and measured using ImageJ v1.8.0 software.

For the goblet cell numbers, Carnoy’s-fixed sections (60% methanol, 30% chloroform, and 10% acetic acid) were stained with the Alcian blue-periodic acid Schiff (AB-PAS) following the manufacturer’s instruction (Solarbio, Beijing, China). Briefly, sections were stained with 1% Alcian blue solution (pH 2.5) in 3% acetic acid solution for 15–20min. The slides were then rinsed in distilled water for 2min and oxidized in 0.5% periodic acid solution for 5min. Slides were rinsed and stained in Schiff reagent for 15min, followed by hematoxylin staining. The measurements of the stained slides were carried out with a DM300 light microscope (Leica, Germany) at 100 magnification. The number of goblet cells was counted manually in 10 colonic crypts per section.

For the mucus thickness, Carnoy’s fixed sections were deparaffinized, rehydrated, and followed by blocked in carbon-free blocking solution (SP-5040, Vector Laboratories) for 1h at room temperature. All sections were then incubated in FITC conjugated UEA-I lectin for 1h at room temperature. After washing three times in PBS, the sections were counterstained with DAPI and analyzed by confocal laser scanning microscopy (Leica TCS SP8, Germany). For determination of mucus thickness using UEA-stained tissue sections, 6 measurements were taken from each colon. Each measurement, the vertical distance between the epithelial cell surface and the luminal mucus surface was calculated using ImageJ v1.8.0 software.

### Western blot analysis

The total protein was extracted from colon tissues using RIPA lysis buffer and quantified using a bicinchoninic acid (BCA) protein assay kit (Thermo Fisher Scientific, MA, USA). Equal amounts of protein were loaded on sodium dodecyl sulfate-polyacrylamide gel electrophoresis gel and then blotted to polyvinylidene difluoride membrane. Membranes were blocked with 5% skimmed milk for 2h and overnight incubated with primary antibodies at 4°C, followed by incubation with HRP-conjugated secondary antibody (Abcam, #ab6721, #ab6789) for 2h at room temperature. Protein signals were detected with ECL kit (Bio-Rad, CA, USA) and visualized using Bio-Rad Chemi XRS imaging system (Bio-Rad). The primary antibodies used were purchased from the following suppliers: anti-β-actin (Proteintech, #20536-1-AP), anti-ZO-1 (Thermo Fisher Scientific Inc., #61-7300), anti-Occludin (Thermo Fisher Scientific Inc., #40-4700), anti-Claudin1 (Thermo Fisher Scientific Inc., #51-9000), anti-NLRP3 (Proteintech, #19771-1-AP), anti-ASC (Proteintech, #10500-1-AP), anti-Caspase1 (Santa Cruz, #14F468), anti-ATG5 (Proteintech, #10182-2-AP), anti-ATG7 (Proteintech, #10088-2-AP), anti-p62 (Cell Signaling Technology, #5114), and anti-LC3 I/II (Affinity Biosciences, #AF5402). Band density of target protein was quantified after normalization to β-actin using ImageJ v1.8.0 software.

### Bacterial translocation

The primer sequences of *E. coli* and 16S were shown in Table S6. PCR products were ligated to the pMD18-T vector system (Takara, Japan), which was subsequently transformed into *E. coli* strain DH5α. Plasmid DNA carrying the insert was extracted and used as the template for DNA sequencing. Ten-fold serial dilutions of plasmid pMD18-T from 10^-3^ to 10^-8^ or 10^-3^ to 10^-6^ were performed to generate standard curve for absolute quantification. Microbial DNA was extracted from the spleen, liver, and colon using the EZNA ^TM^ Soil DNA kit (Omega Bio-Tek Inc., GA, USA) according to the manufacturer’s protocol. qPCR was performed to quantify *E. coli* on an ABI 7300 real-time PCR system (Applied Biosystems, USA). The 20-μL PCR reaction mixture contained 10 μL ChamQ SYBR Color qPCR Master Mix (2×), 0.8 μL of each primer, 0.4 μL ROX Reference Dye 1 (50×), and 2 μL of the template DNA. The cycling conditions were as follows: 95°C for 3 min, followed by 40 cycles at 95°C for 5 s, 58°C for 30 s, and 72°C for 1 min.

### Quantitative analysis of metabolites

SCFAs in the colonic content and feces were quantified using gas chromatography (GC) based on our previous study [85]. Briefly, the digesta or feces were dissolved in distilled water and then centrifuged at 9000 g for 10min. Metaphosphoric acid (25%, w/v) was added into the supernatants at a ratio of 1:9. After centrifugation at 10,000 g, the supernatants were filtered through a 0.45-μm membrane and then subjected to GC system (Agilent 6890N, Palo Alto, CA). The lactate concentration in the colonic contents was determined by the commercial kit following the manufacturer’s protocol (Nanjing Jiancheng Bioengineering Institute, Nanjing, China).

A total of 17 BAs in the colon and feces were profiled with a Waters Xevo TQ-S LC/MS mass spectrometers (Waters, Milford, MA, USA) equipped with an ESI source according to our previous reported by Fang et al. [86]. In brief, the lyophilized digesta or feces were suspended in pre-cold sodium acetate buffer (50 mM, pH 5.6)/ethanol (v/v=1:3), and then, the mixture was vortexed for 2min to mix thoroughly. After centrifugation at 20,000 g for 20min, the supernatant was diluted five times with sodium acetate buffer and applied to a Bond Elute C18 cartridge (500 mg/6mL, Agilent). The cartridge was eluted with 5 mL methanol. The residue was dissolved in 1 mL methanol after the eluent was evaporated with nitrogen gas and finally filtered with a 0.45-μm membrane. The BAs were separated on a ZORBAX Eclipse plus C18 column (95Å, 1.8 μm, 2.1 ×100 mm) and eluted using a gradient of water and acetonitrile (ACN) with 0.1% formic acid at a flow rate of 0.4 mL/min. The spray voltage and vaporizer temperature were set at 2.91 kV and 500°C, respectively. All BA standards were purchased from Sigma-Aldrich (Darmstadt, Germany). The quantification of each BA was based on the series dilutions of available standards, and good linearity was confirmed.

### 16s rRNA gene high-throughput sequencing

Total bacterial genomic DNA was extracted from the colonic mucosa, contents, or fecal culture samples using the Qiagen DNA isolation kit (Qiagen, Hilden, Germany). The V3-V4 region of bacterial 16S rRNA was amplified using primers 338F (5′- ACTCCTACGGGAGGCAGCAG-3′) and 806R (5′-GGACTACHVGGGTWTCTAAT-3′). Sequencing of the PCR amplification products was performed on the Illumina MiSeq sequencing platform. Sequence data were analyzed with Quantitative Into Microbial Ecology (QIIME) package version 1.9.1., using the Silva 138 reference database (https://www.arb-silva.de/) as a reference template. Low abundant OTUs were removed by filtering OTUs that had <10% of samples below 10 read counts. The principal coordinate analysis (PCoA) based on Bray-Curtis distance and LEfSe analysis were conducted on the free online platform of Majorbio Cloud Platform (www.majorbio.com). Statistical differential taxa between two groups were calculated by the Mann-Whitney *U* test (nonparametric test) with *P* < 0.05 and the threshold of LDA score with 3.0.

### Mucin extraction, O-glycan release, and characterization

The isolation of colonic mucins was obtained following a previous study [10]. Briefly, after remove contents, adherent mucus in the colon was gently scraped off with a glass slide. Mucus was placed in a microtube along with a complete protease inhibitors cocktail, and five volumes of guanidium chloride extraction buffer (6 M GuHCl, 0.1 M Tris, 1 mM EDTA, pH 8.0) were added. The mixture was dispersed with a homogenizer and then extracted overnight at 4°C on a rotator. The secretions were centrifuged by 20,000 g for 40min at 4°C, and then, the supernatant was collected. The pellets were redissolved in five volumes of guanidium chloride extraction buffer and extracted with three repeat times. Finally, the extracts were pooled. Following extraction, samples were reduced with 100 mM dithiothreitol (DTT) for 5h at 37°C. After then, 100 mM DTT was again added to solubilize the gel-forming mucin. The mucus was alkylated with 250 mM iodoacetamide overnight in the dark at room temperature. The mucins were then dialyzed against ddH_2_O and lyophilized.

Mucin *O*-glycans were released by β-elimination and subjected to PGC-LC-ESI-MS/MS analysis according to a previous report [87]. Briefly, mucins were reduced with 0.5 M sodium borohydride and 50 mM sodium hydroxide for 16h at 50°C to release *O*-glycans. The reduction reaction was quenched by the addition of glacial acetic acid and desalted by a cation exchange column (Bio-Rad, CA, USA). Excess borate was removed by adding methanol five times. Finally, the purified *O*-glycans were resuspended in 10 mM NH_4_CO_3_. *O*-glycans were separated on a graphitized carbon column (250Å, 2.1 mm × 100 mm, 3 μm) connected to a 6600 Q-TOF mass spectrometer (AB SCIEX) and eluted using a gradient water and ACN with 10 mM NH_4_CO_3_ at a flow rate of 7 μL/min. The MS and MS/MS spectra were acquired under the negative ion mode with an electrospray voltage of 3.5 kV and capillary temperature of 300°C. The GlycoMod Tool on the ExPASy Server (https://web.expasy.org/glycomod/) was used to match MS masses with glycan compositions. Glycan structures were assigned by manual interpretation of the tandem MS fragmentation spectra. Glycan peaks were quantified by relative abundance using ProteoWizard project (https://proteowizard.sourceforge.io/) for assisted peak picking and integration of peak areas. Glycan structures were drawn using GlycoWorkbench 2 based on SNFG nomenclature.

### Statistical analysis

The obtained data were analyzed using Student’s *t* test or Mann-Whitney *U* test, and one-way analysis of variance (ANOVA) followed by least significant difference post hoc tests for multiple groups using JMP 13.0 (SAS Institute, Cary, NC, USA) or R program (https://www.r-project.org/). Significance was defined when the *P* value was <0.05.

Partial least squares discriminant analysis (PLS-DA) and DIABLO were performed using the package “mixOmics” in R program. Mantel’s correlation was applied using the package “ggcor” in R program.

## Supplementary Information

**Additional file 1: Figure S1**. Diarrheal piglets exhibit colonic microbiota dysbiosis. Weighted UniFrac PCoA plots of (A) the colonic mucosal and (B) luminal microbiota composition. Chao1 index of (C) mucosa and (G) lumen. Shannon index of (D) mucosa and (H) lumen. Simpson index of (E) mucosa and (I) lumen. ACE index of (F) mucosa and (J) lumen. Cladogram representing taxa (LDA score ≥ 2) enriched in (K) colonic mucosa and (L) lumen. Data are presented as min to max. H: Healthy controls; D: Diarrheal piglets; PCoA: Principal coordinate analysis; LDA: linear discriminant analysis.

**Additional file 2: Figure S2**. Bacteria-derived metabolites levels in piglets. The concentrations of (A) total SCFAs, (B) acetate, (C) propionate, (D) isobutyrate, (E) butyrate, (F) isovalerate, (G) valerate, (H) lactate. The composition of each BA in the (I) colonic contents and (J) feces of piglets. The concentrations of (K) PBA, (L) SBA, (M) TCBA, and (N) GCBA. Data are presented as min to max showing all points. H: Healthy controls; D: Diarrheal piglets; SCFAs: Short chain fatty acids; BA: Bile acid; PBA: Primary bile acids; SBA: Secondary bile acids; TCBA: Taurine-conjugated bile acids; GCBA: Glycine-conjugated bile acids; TCA: Taurocholic acid; TCDCA: Taurochenodeoxycholic acid; GCA: Glycocholic acid; GCDCA: Glycochenodeoxycholic acid; TLCA: Taurolithocholic acid; TUCDA: Tauroursodeoxycholic acid; GDCA: Glycodeoxycholic acid; GUDCA: Glycoursodeoxycholic acid; THDCA: Taurohyodeoxycholic acid; TDCA: Taurodeoxycholic acid; UDCA: Ursodeoxycholic acid.

**Additional file 3: Figure S3**. Bile acid profiles in the colonic contents and feces. The concentrations of (A) CA, (B) CDCA, (C) HCA, (D) DCA, (E) LCA, (F) HDCA, (G) UDCA, (H) TCA, (I) TCDCA, (J) TLCA, (K) TUDCA, (L) THDCA, (M) TDCA, (N) GDCA, (O) GUDCA, (P) GCA, and (Q) GCDCA. Data are presented as relative percentage or min to max showing all points. H: healthy controls; D: Diarrheal piglets.

**Additional file 4: Figure S4**. Pathophysiological features of diarrhea in piglets. (A) Body weight; The (B) colon and (C) total gut length; (D) The colon-to-total gut length ratio; Representative images of (E) liver and (G) spleen; (F) Liver weight and (H) spleen weight. Data are presented as mean ± SE. H: Healthy controls; D: Diarrheal piglets.

**Additional file 5: Figure S5**. Expression of glycosyltranferases in piglets. (A) The mRNA levels of FUT1, FUT2, GAL3ST1, GAL3ST2, and ST6GAL1 in the colon of piglets. (B) The mRNA levels of glycosyltransferases related with elongation of the glycan structures: *GCNT2*, *B3GNT3*, *B3GALT5*, *B4GALT4*, *B4GALT5*, *B4GALT7*, and *B4GALT12* in the colon of piglets. Data are presented as mean ± SE. H: healthy controls; D: Diarrheal piglets.

**Additional file 6: Figure S6**. Inflammatory response in piglets. The levels of IL-10 in (A) serum and (B) colon. The levels of (C) IL-1β and (D) IFN-γ in serum. (E) The copy number of total bacteria in the colonic mucosa. (F) Circos plots displaying correlations between the discriminant OTUs, index related with intestinal barrier function, and metabolites. Positive and negative correlations (r>0.6) were displayed by red and blue links, respectively. Data are presented as mean ± SE showing all points. H: Healthy controls; D: Diarrheal piglets.

**Additional file 7: Figure S7**. Effect of colonic mucin glycans on SCFAs production and alpha diversity of the microbiota. The concentrations of (A) acetate, (B) propionate, (C) isobutyrate, (D) butyrate, (E) isovalerate, (F) valerate. The (G) Chao1, (H) Shannon, (I) Simpson, and (J) ACE indexes of colonic mucins fermentation *in vitro*. Data are presented as min to max showing all points. H: healthy controls; D: Diarrheal piglets.

**Additional file 8: Table S1**. Colon taxa altered in the mucosa and lumen of piglets.

**Additional file 9: Table S2**. Structures and relative abundance of *O*-glycans from colonic mucins in piglets.

**Additional file 10: Table S3**. Differentially abundant taxa between mucins from diarrheal piglet and healthy control fermentation.

**Additional file 11: Table S4**. The standard of fecal rating.

**Additional file 12: Table S5**. The information of piglets.

**Additional file 13: Table S6**. Primer sequences used for qPCR in this study.

## Data Availability

The sequences generated in this study are available in the NCBI Sequence Read Archive database (Accession Number: PRJNA773865).

## Abbreviations

AB-PAS: Alcian blue-periodic acid-Schiff
AKP: Alkline phosphatase
AMPK: Adenosine monophosphate-activated protein kinase
BAs: Bile acids
CMGs: Colonic mucin glycans
DAO: Diamine oxidase
DIABLO: Data Integration Analysis for Biomarker discovery using a Latent component method for Omics
GalNAc: *N*-acetyl-galactosamine
GALNT: GalNAc-transferase
GlcNAc: *N*-acetyl-glucosamine
IBD: Inflammatory bowel disease
LC-MS/MS: Liquid chromatography-tandem mass spectrometry
LEfSe: Linear discriminant analysis effect size
PBA: Primary bile acids
PCoA: Principal coordinates analysis
PLS-DA: Partial least squares discriminant analysis
SBA: Secondary bile acids
SCFAs: Short-chain fatty acids
