# Supplementary figures and images for: Mucin O-glycan-microbiota axis orchestrates gut homeostasis in a diarrheal pig model
Source: Microbiome. 2022 Aug 31;10:139. doi: 10.1186/s40168-022-01326-8 (PMC9429786; doi:10.1186/s40168-022-01326-8)

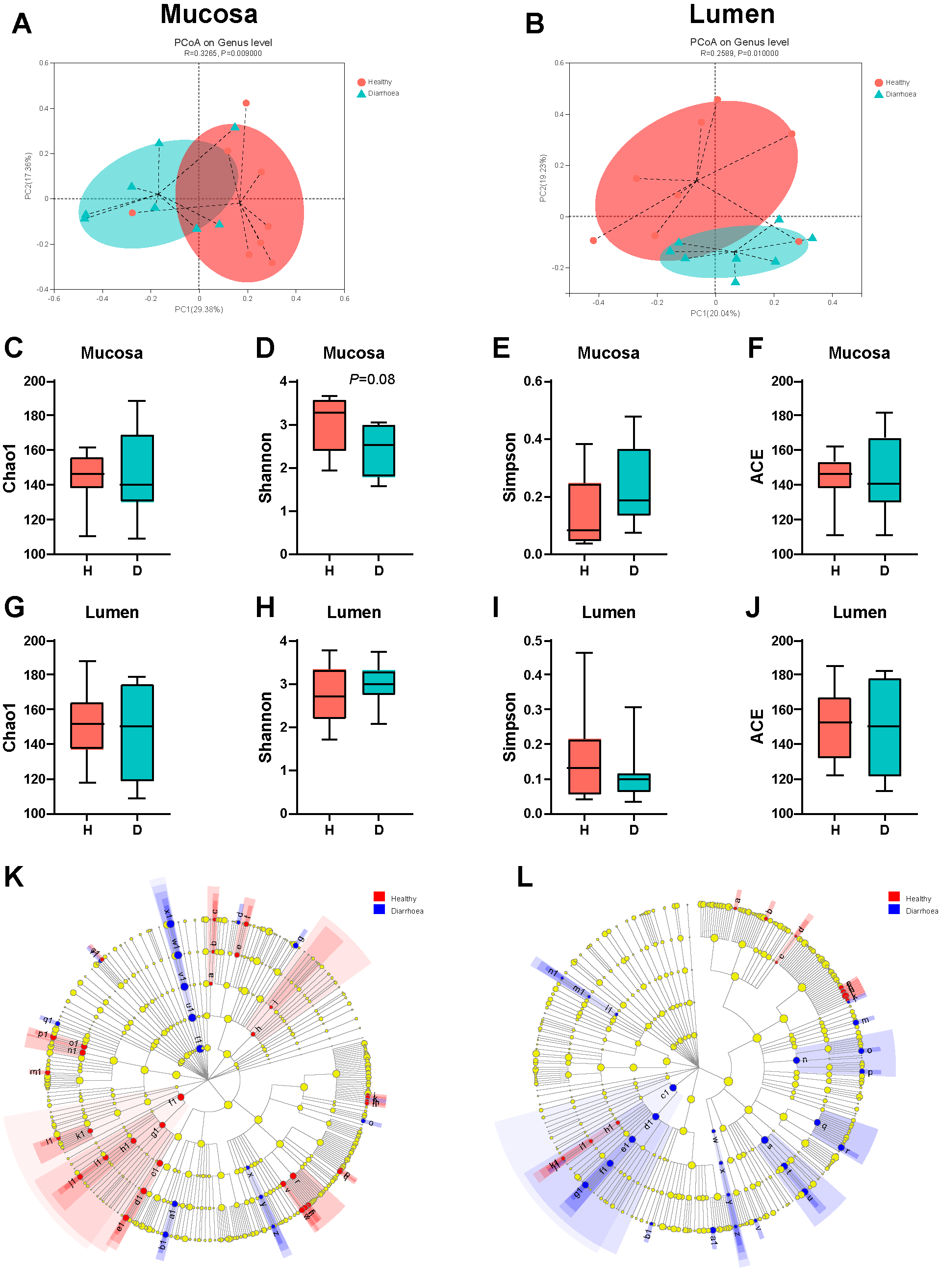

Supplement: Supplementary file 2 — Additional file 1: Figure S1. Diarrheal piglets exhibit colonic microbiota dysbiosis. Weighted UniFrac PCoA plots of (A) the colonic mucosal and (B) luminal microbiota composition. Chao1 index of (C) mucosa and (G) lumen. Shannon index of (D) mucosa and (H) lumen. Simpson index of (E) mucosa and (I) lumen. ACE index of (F) mucosa and (J) lumen. Cladogram representing taxa (LDA score ≥ 2) enriched in (K) colonic mucosa and (L) lumen. Data are presented as min to max. H: Healthy controls; D: Diarrheal piglets; PCoA: Principal coordinate analysis; LDA: linear discriminant analysis. [file 40168_2022_1326_MOESM1_ESM.tif]

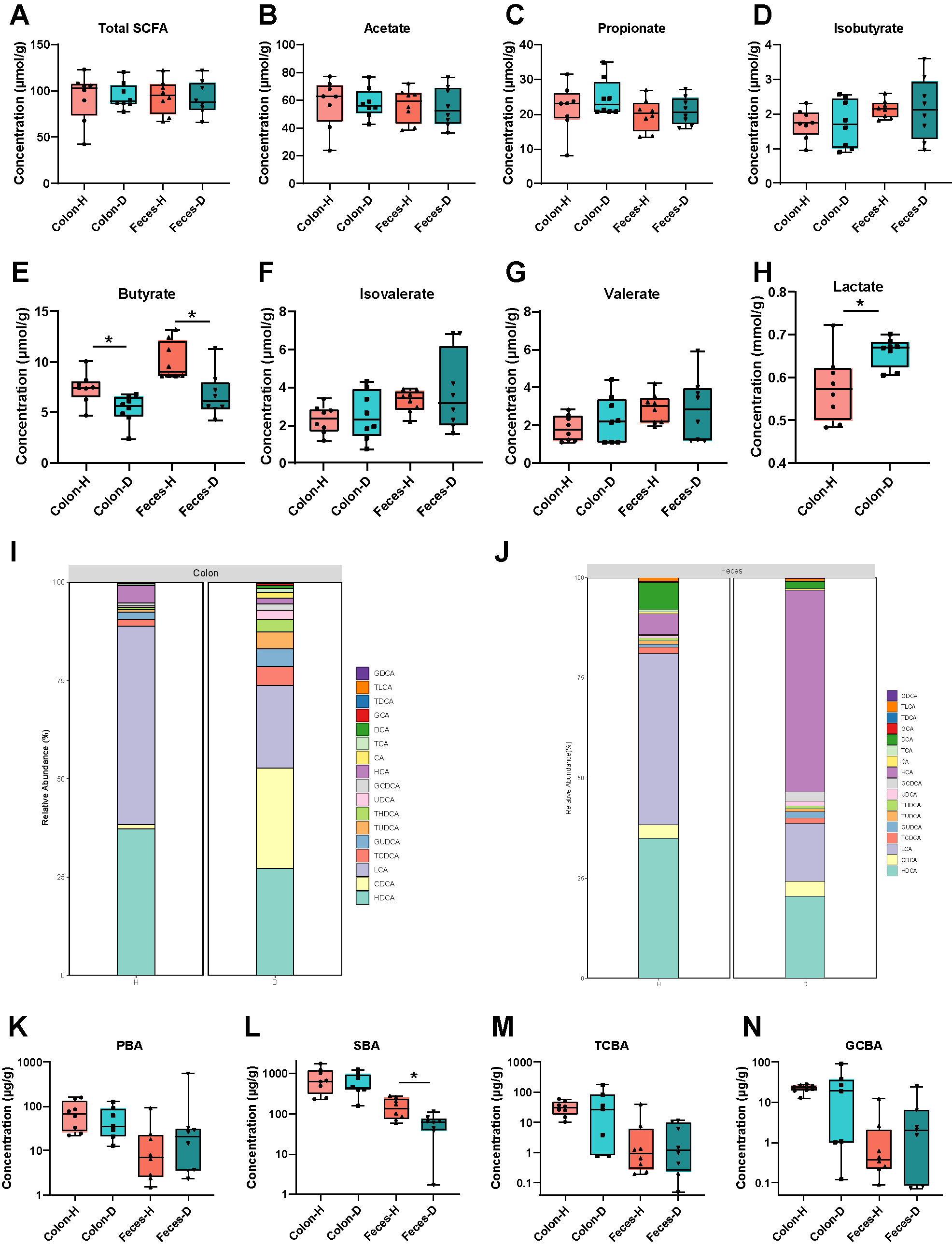

Supplement: Supplementary file 3 — Additional file 2: Figure S2. Bacteria-derived metabolites levels in piglets. The concentrations of (A) total SCFAs, (B) acetate, (C) propionate, (D) isobutyrate, (E) butyrate, (F) isovalerate, (G) valerate, (H) lactate. The composition of each BA in the (I) colonic contents and (J) feces of piglets. The concentrations of (K) PBA, (L) SBA, (M) TCBA, and (N) GCBA. Data are presented as min to max showing all points. H: Healthy controls; D: Diarrheal piglets; SCFAs: Short chain fatty acids; BA: Bile acid; PBA: Primary bile acids; SBA: Secondary bile acids; TCBA: Taurine-conjugated bile acids; GCBA: Glycine-conjugated bile acids; TCA: Taurocholic acid; TCDCA: Taurochenodeoxycholic acid; GCA: Glycocholic acid; GCDCA: Glycochenodeoxycholic acid; TLCA: Taurolithocholic acid; TUCDA: Tauroursodeoxycholic acid; GDCA: Glycodeoxycholic acid; GUDCA: Glycoursodeoxycholic acid; THDCA: Taurohyodeoxycholic acid; TDCA: Taurodeoxycholic acid; UDCA: Ursodeoxycholic acid. [file 40168_2022_1326_MOESM2_ESM.tif]

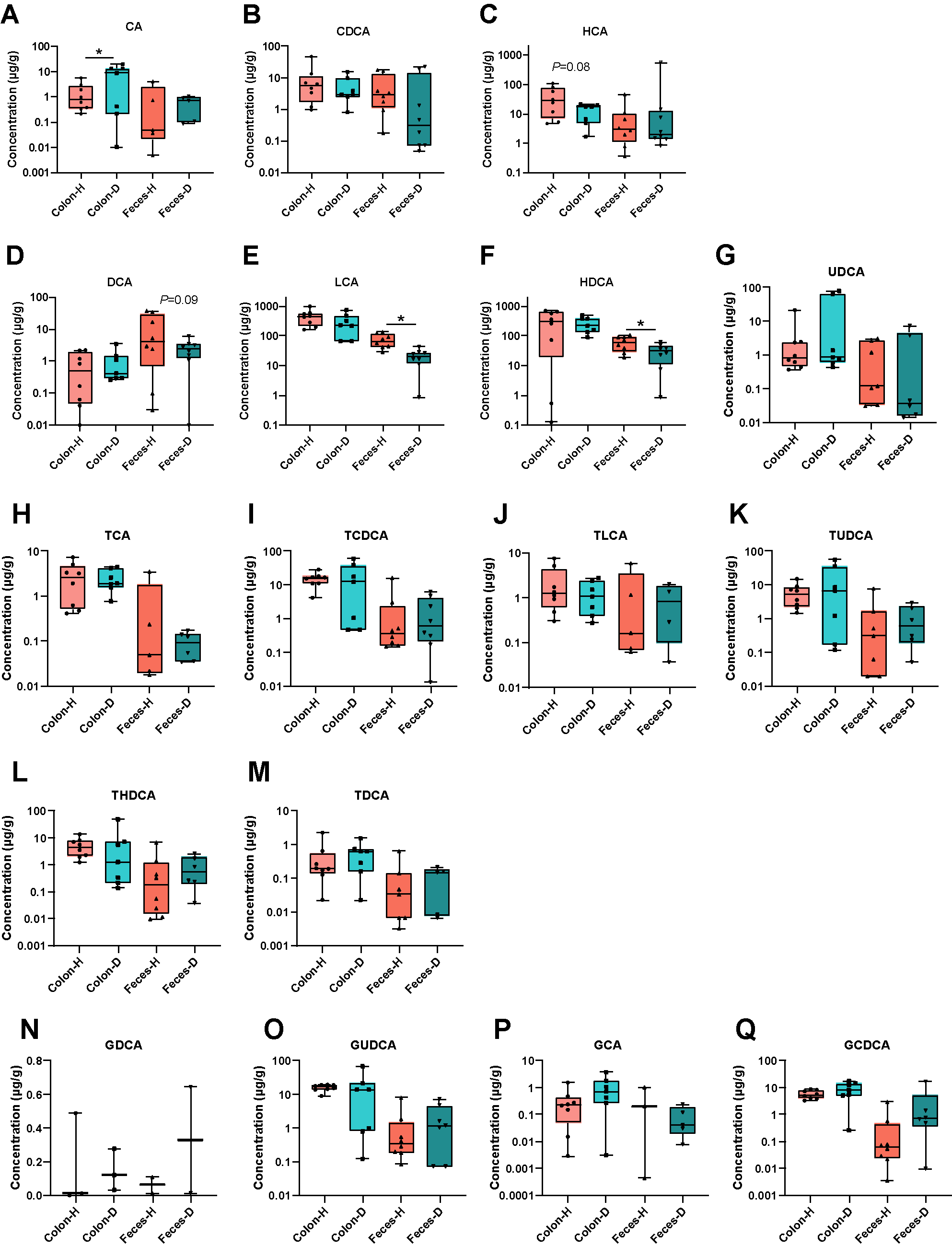

Supplement: Supplementary file 4 — Additional file 3: Figure S3. Bile acid profiles in the colonic contents and feces. The concentrations of (A) CA, (B) CDCA, (C) HCA, (D) DCA, (E) LCA, (F) HDCA, (G) UDCA, (H) TCA, (I) TCDCA, (J) TLCA, (K) TUDCA, (L) THDCA, (M) TDCA, (N) GDCA, (O) GUDCA, (P) GCA, and (Q) GCDCA. Data are presented as relative percentage or min to max showing all points. H: healthy controls; D: Diarrheal piglets. [file 40168_2022_1326_MOESM3_ESM.tif]

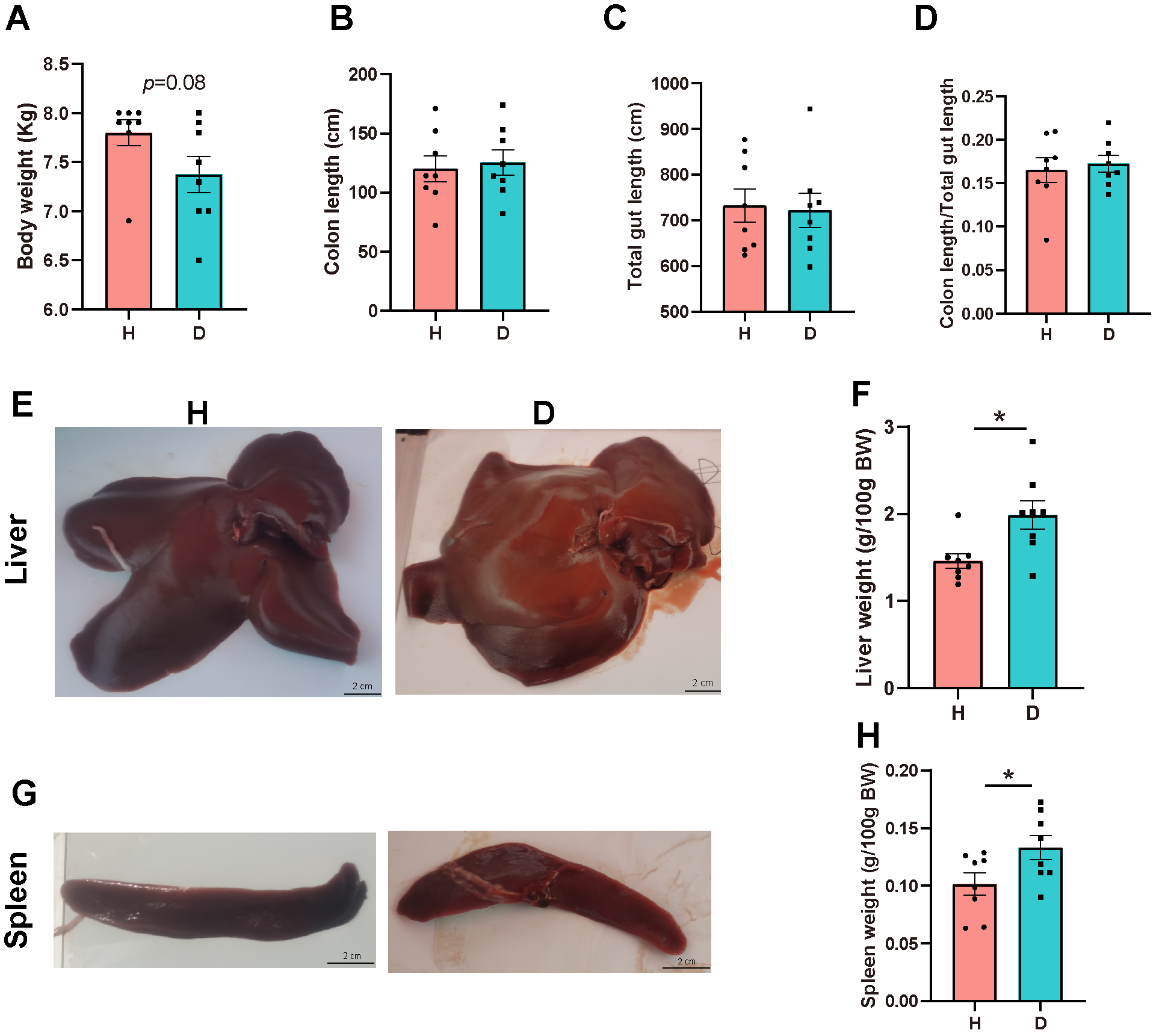

Supplement: Supplementary file 5 — Additional file 4: Figure S4. Pathophysiological features of diarrhea in piglets. (A) Body weight; The (B) colon and (C) total gut length; (D) The colon-to-total gut length ratio; Representative images of (E) liver and (G) spleen; (F) Liver weight and (H) spleen weight. Data are presented as mean ± SE. H: Healthy controls; D: Diarrheal piglets. [file 40168_2022_1326_MOESM4_ESM.tif]

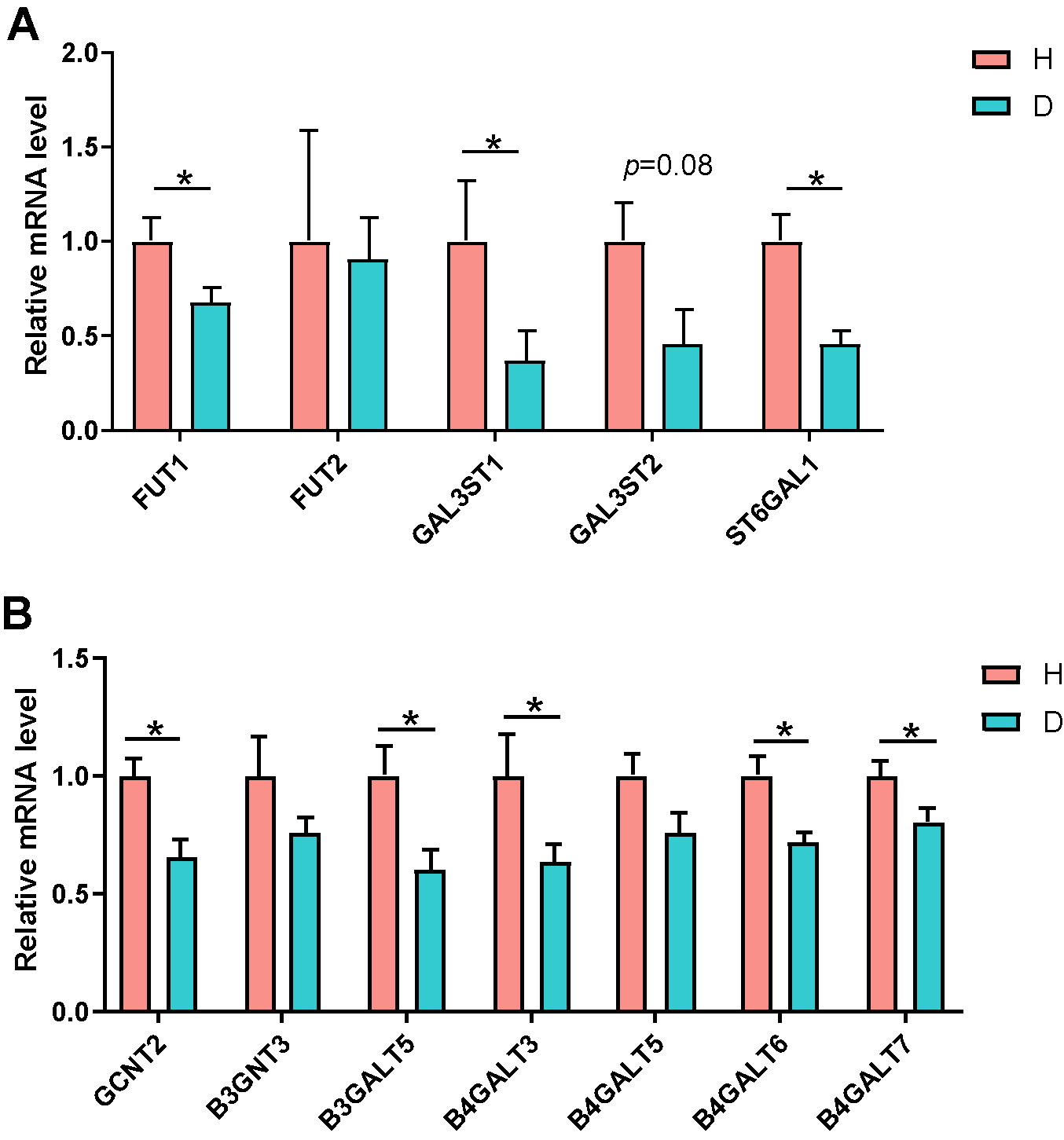

Supplement: Supplementary file 6 — Additional file 5: Figure S5. Expression of glycosyltranferases in piglets. (A) The mRNA levels of FUT1, FUT2, GAL3ST1, GAL3ST2, and ST6GAL1 in the colon of piglets. (B) The mRNA levels of glycosyltransferases related with elongation of the glycan structures: GCNT2, B3GNT3, B3GALT5, B4GALT4, B4GALT5, B4GALT7, and B4GALT12 in the colon of piglets. Data are presented as mean ± SE. H: healthy controls; D: Diarrheal piglets. [file 40168_2022_1326_MOESM5_ESM.tif]

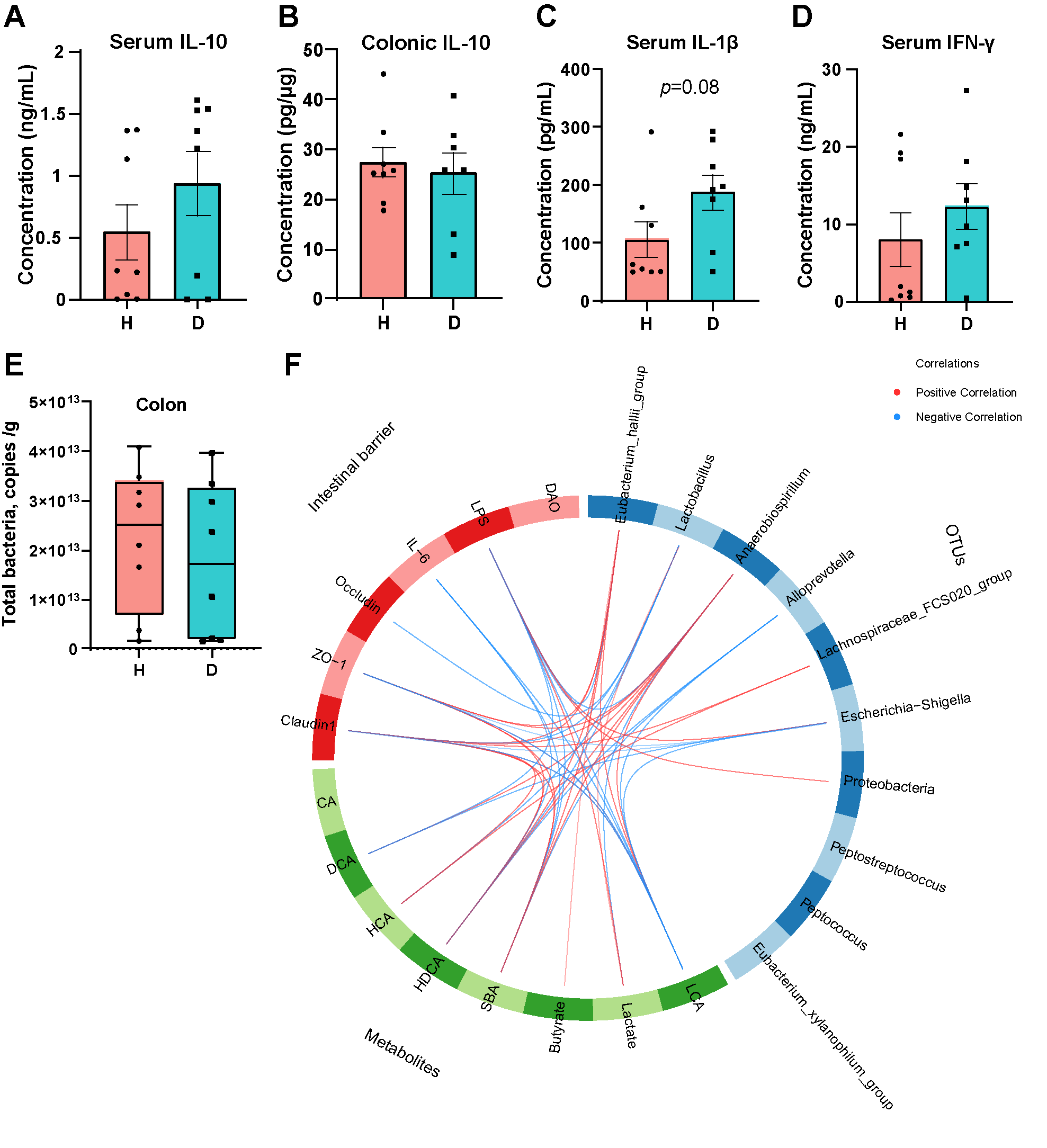

Supplement: Supplementary file 7 — Additional file 6: Figure S6. Inflammatory response in piglets. The levels of IL-10 in (A) serum and (B) colon. The levels of (C) IL-1β and (D) IFN-γ in serum. (E) The copy number of total bacteria in the colonic mucosa. (F) Circos plots displaying correlations between the discriminant OTUs, index related with intestinal barrier function, and metabolites. Positive and negative correlations (r>0.6) were displayed by red and blue links, respectively. Data are presented as mean ± SE showing all points. H: Healthy controls; D: Diarrheal piglets. [file 40168_2022_1326_MOESM6_ESM.tif]

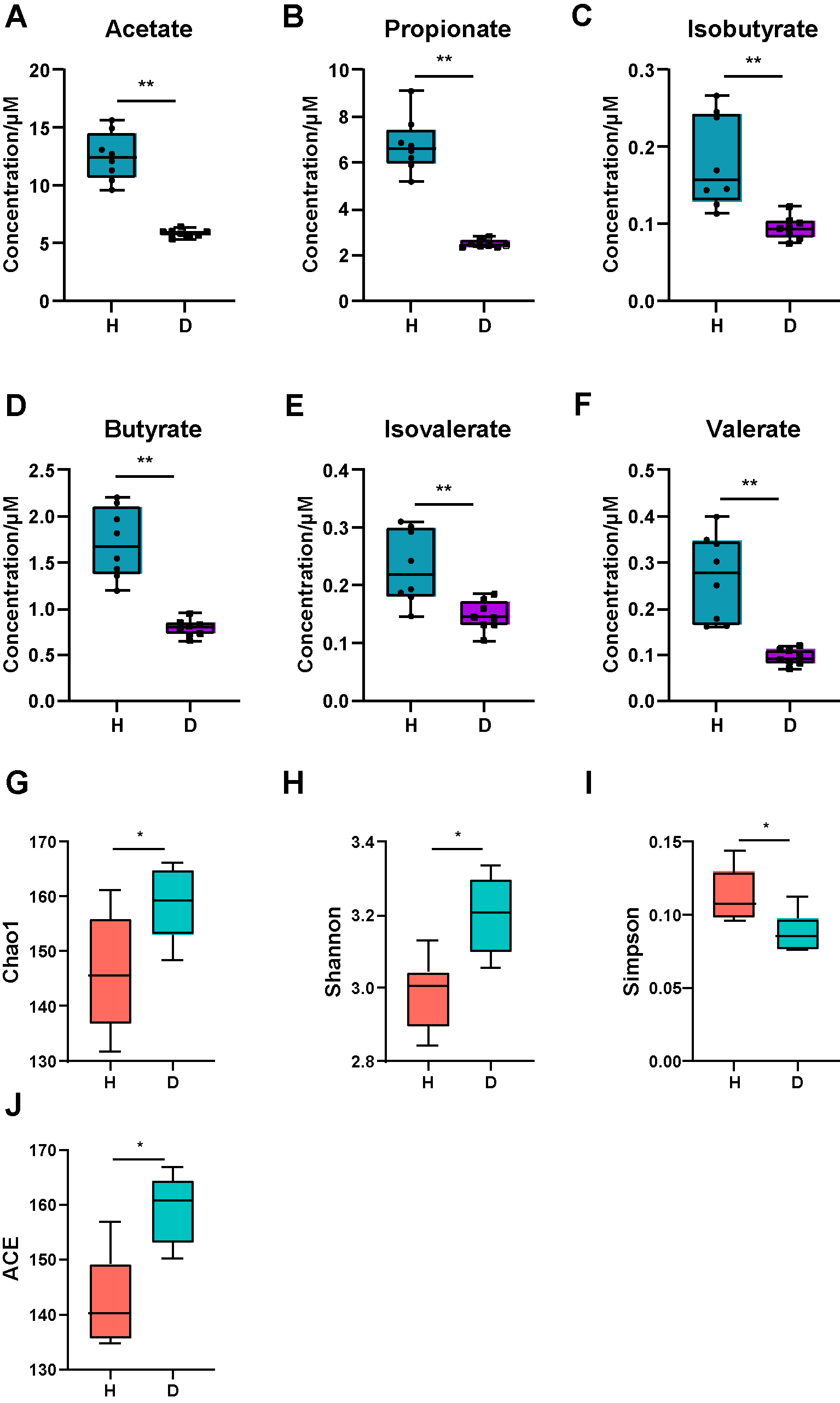

Supplement: Supplementary file 8 — Additional file 7: Figure S7. Effect of colonic mucin glycans on SCFAs production and alpha diversity of the microbiota. The concentrations of (A) acetate, (B) propionate, (C) isobutyrate, (D) butyrate, (E) isovalerate, (F) valerate. The (G) Chao1, (H) Shannon, (I) Simpson, and (J) ACE indexes of colonic mucins fermentation in vitro. Data are presented as min to max showing all points. H: healthy controls; D: Diarrheal piglets. [file 40168_2022_1326_MOESM7_ESM.tif]
