# Supplementary material for: Mucin O-glycan-microbiota axis orchestrates gut homeostasis in a diarrheal pig model
Source: Microbiome. 2022 Aug 31;10:139. doi: 10.1186/s40168-022-01326-8 (PMC9429786; doi:10.1186/s40168-022-01326-8)
Supplement: Supplementary file 10 — Additional file 9: Table S2. Structures and relative abundance of O-glycans from colonic mucins in piglets. [file 40168_2022_1326_MOESM9_ESM.docx]

**Table S2 Structures and relative abundance of *O*-glycans from colonic mucins in piglets**

| **Structure** | **Mass (m/z)** | | **Core** | **Mean±SE** | |
| --- | --- | --- | --- | --- | --- |
|  | **M-H** | **M** |  | **H** | **D** |
| 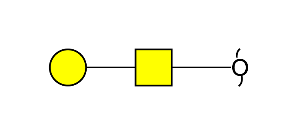 | 384 | 385 | 1 | 1.32±0.19 | 2.17±0.22 |
| 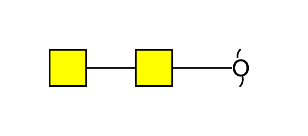 | 425 | 426 | 3 | 2.15±0.35 | 2.98±0.32 |
| 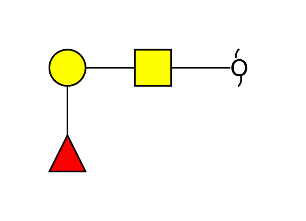 | 530 | 531 | 1 | 2.63±0.33 | 3.68±0.54 |
| 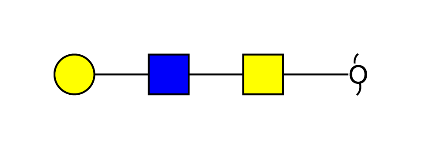 | 587a | 588a | 3 | 1.36±0.27 | 1.20±0.40 |
| 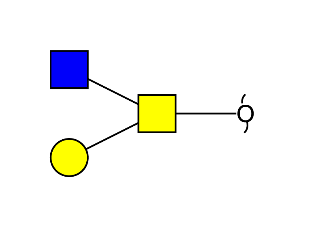 | 587b | 588b | 2 | 1.19±0.24 | 1.90±0.28 |
| 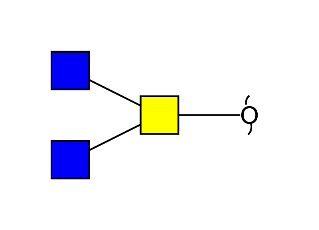 | 628 | 629 | 4 | 1.66±0.31 | 2.00±0.17 |
| 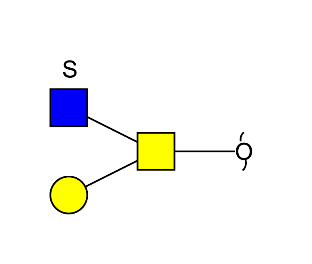 | 667 | 668 | 2 | 5.24±0.80 | 6.02±0.76 |
| 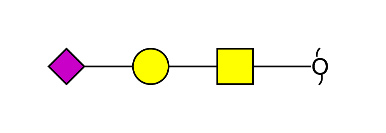 | 675a | 676a | 1 | 2.89±0.54 | 2.11±0.60 |
| 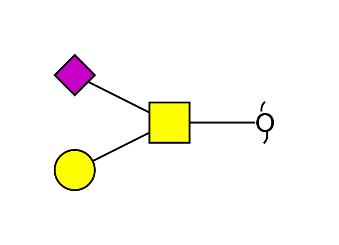 | 675b | 676b | 1 | 2.27±0.39 | 3.61±0.70 |
| 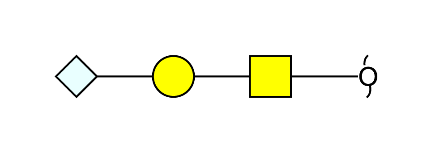 | 691a | 691a | 1 | 1.33±0.44 | 0.49±0.35 |
| 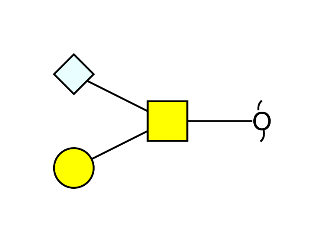 | 691b | 691b | 1 | 1.71±0.26 | 0.63±0.28 |
| 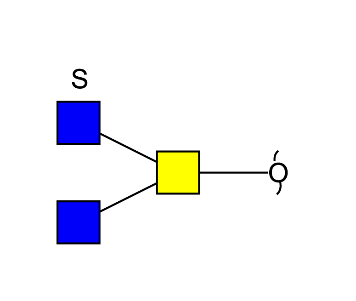 | 708 | 709 | 4 | 5.24±0.65 | 5.95±1.45 |
| 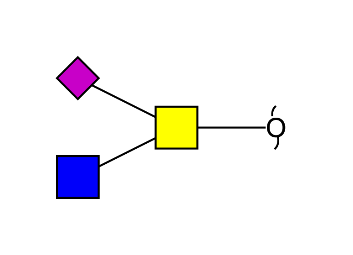 | 716a | 717a | 3 | 0.77±0.25 | 1.13±0.44 |
| 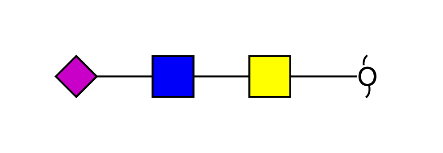 | 716b | 717b | 3 | 0.88±0.24 | 0.87±0.20 |
| 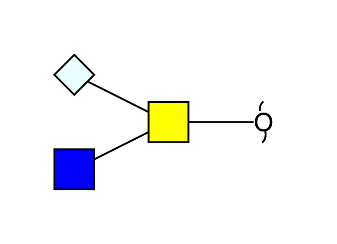 | 732 | 733 | 3 | 1.87±0.29 | 1.39±0.20 |
| 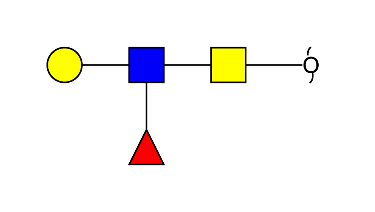 | 733a | 734a | 3 | 1.79±0.42 | 1.35±0.47 |
| 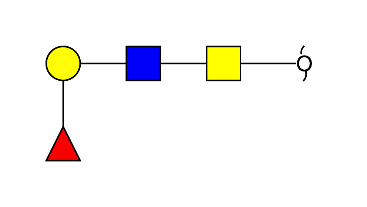 | 733b | 734b | 3 | 1.43±0.23 | 1.59±0.48 |
| 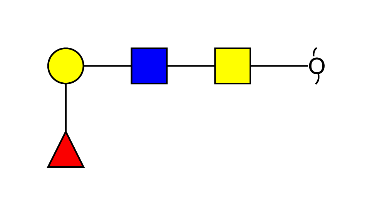 | 733c | 734c | 3 | 0.93±0.24 | 0.44±0.06 |
| 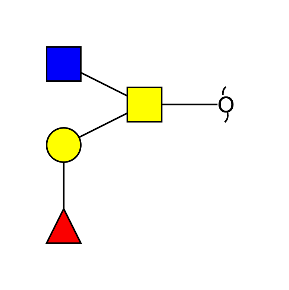 | 733d | 734d | 2 | 0.73±0.10 | 1.00±0.23 |
| 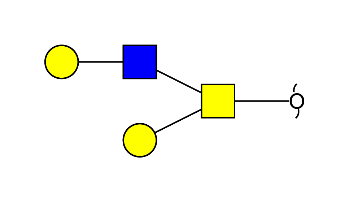 | 749 | 750 | 2 | 1.68±0.26 | 2.62±0.23 |
| 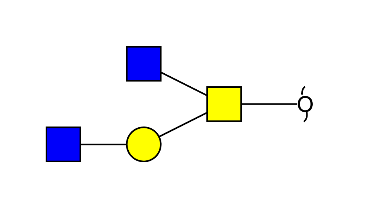 | 790a | 791a | 2 | 0.31±0.06 | 0.66±0.17 |
| 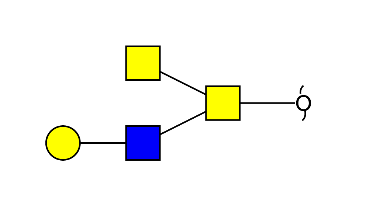 | 790b | 791b | 4 | 4.66±0.85 | 7.04±1.19 |
| 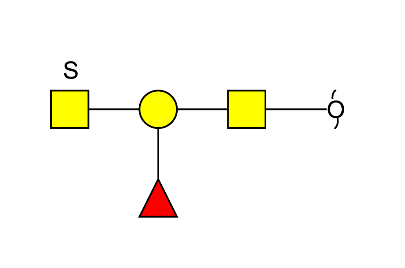 | 813a | 814a | 1 | 0.66±0.06 | 0.45±0.12 |
| 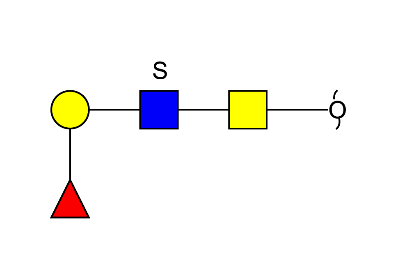 | 813b | 814b | 3 | 5.06±0.97 | 4.27±1.17 |
| 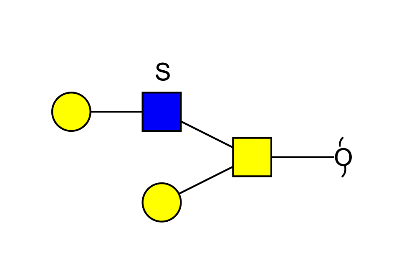 | 829a | 830a | 2 | 3.62±0.42 | 4.06±0.57 |
| 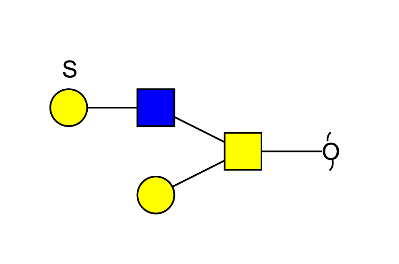 | 829b | 830b | 2 | 1.46±0.38 | 0.56±0.50 |
| 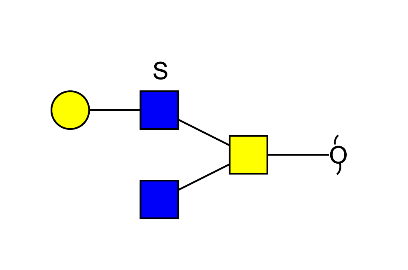 | 870 | 871 | 4 | 1.68±0.20 | 1.36±0.54 |
| 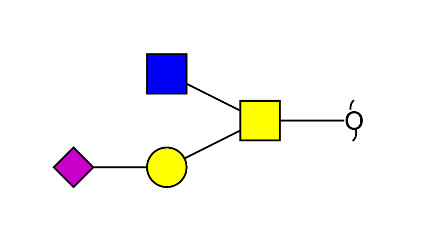 | 878 | 879 | 2 | 0.46±0.06 | 0.35±0.08 |
| 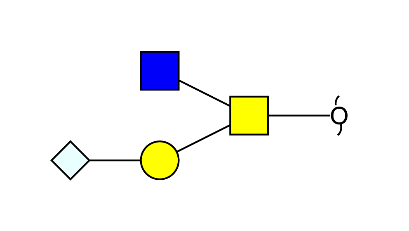 | 894 | 895 | 2 | 0.58±0.06 | 0.56±0.11 |
| 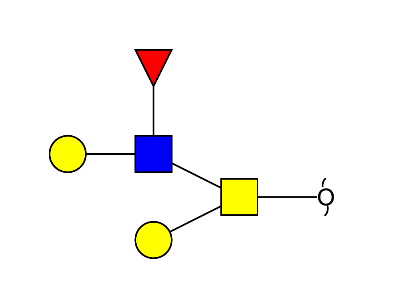 | 895a | 896a | 2 | 0.81±0.23 | 1.23±0.12 |
| 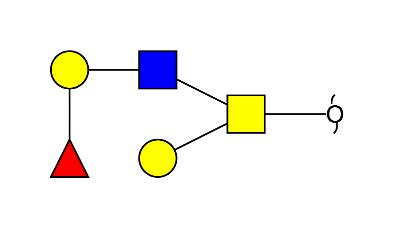 | 895b | 896b | 2 | 0.64±0.09 | 0.79±0.22 |
| 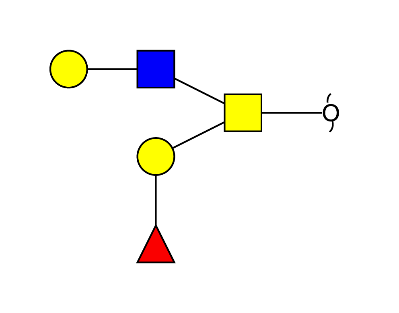 | 895c | 896c | 2 | 0.43±0.04 | 0.32±0.12 |
| 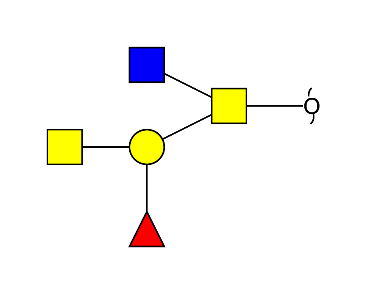 | 936a | 937a | 2 | 3.34±0.52 | 4.97±0.51 |
| 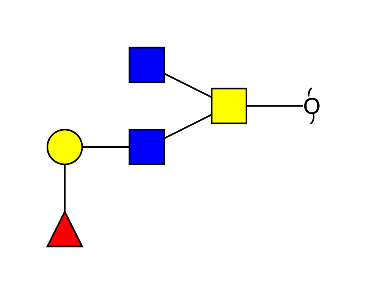 | 936b | 937b | 4 | 0.41±0.13 | 0.92±0.49 |
| 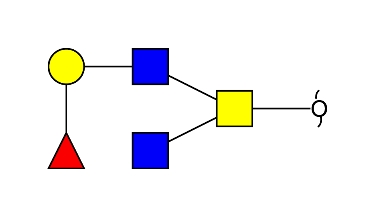 | 936c | 937c | 4 | 2.31±0.22 | 2.84±0.66 |
| 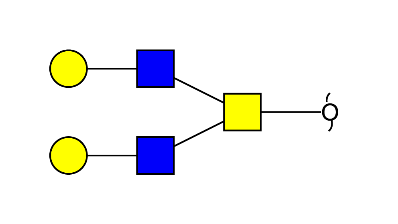 | 952 | 953 | 4 | 3.10±0.57 | 4.75±0.68 |
| 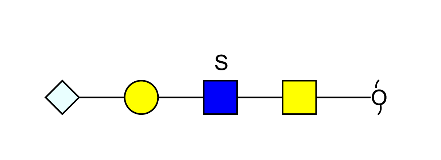 | 974 | 975 | 3 | 0.38±0.06 | 0.04±0.03 |
| 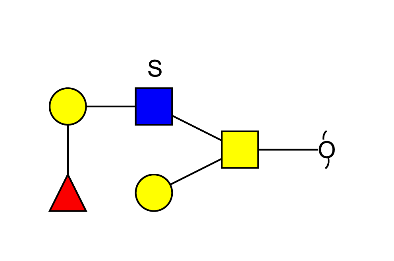 | 975 | 976 | 2 | 2.69±0.46 | 1.19±0.57 |
| 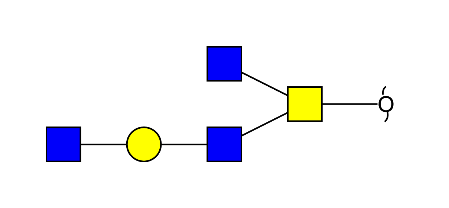 | 993a | 994a | 4 | 0.35±0.08 | 0.18±0.08 |
| 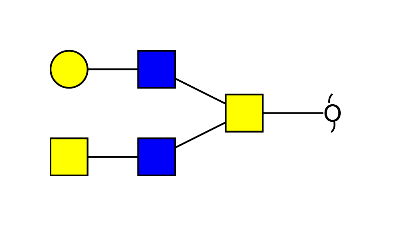 | 993b | 994b | 4 | 0.36±0.08 | 0.38±0.07 |
| 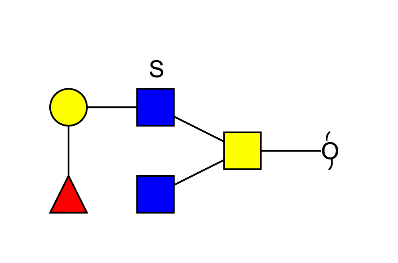 | 1016a | 1017a | 4 | 2.45±0.42 | 2.88±0.88 |
| 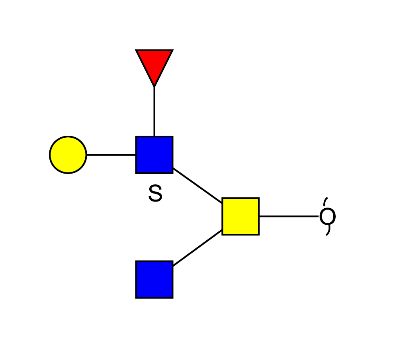 | 1016b | 1017b | 4 | 4.08±1.02 | 2.33±0.71 |
| 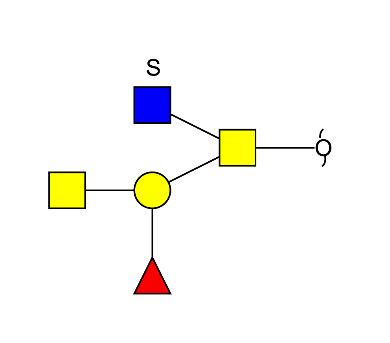 | 1016c | 1017c | 2 | 1.27±0.26 | 1.24±1.16 |
| 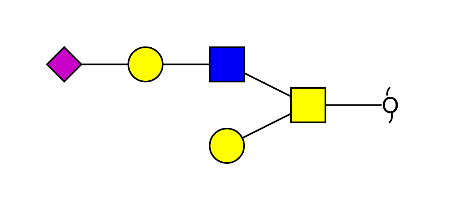 | 1040 | 1041 | 2 | 0.70±0.08 | 0.28±0.14 |
| 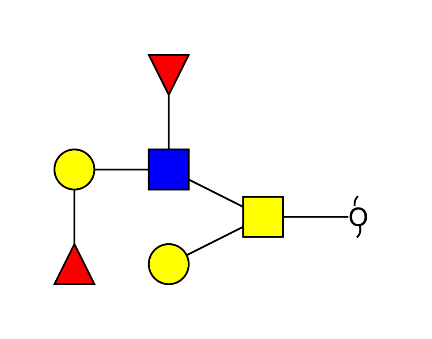 | 1041a | 1042a | 2 | 0.69±0.11 | 0.19±0.17 |
| 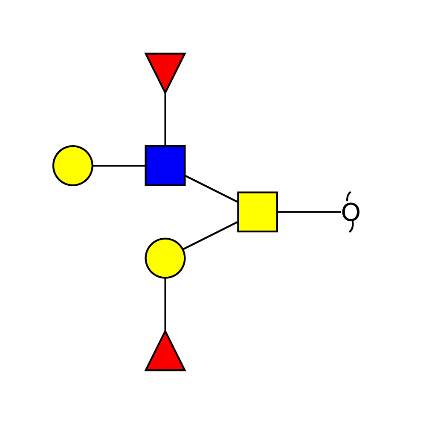 | 1041b | 1042b | 2 | 0.47±0.23 | 0.29±0.27 |
| 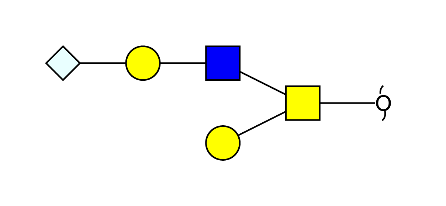 | 1056 | 1057 | 2 | 0.92±0.11 | 0.66±0.27 |
| 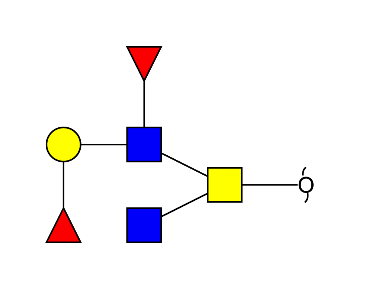 | 1082 | 1083 | 4 | 6.15±0.98 | 4.55±0.62 |
| 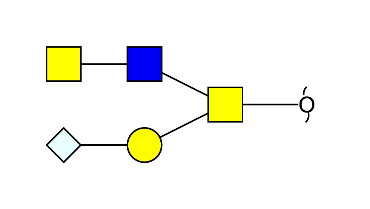 | 1097 | 1098 | 2 | 0.07±0.04 | 0.05±0.03 |
| 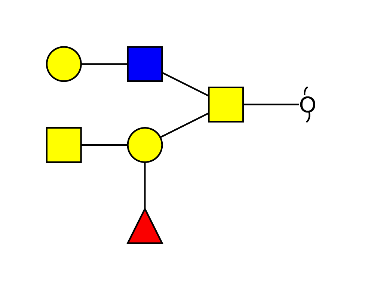 | 1098a | 1099a | 2 | 0.91±0.16 | 1.09±0.20 |
| 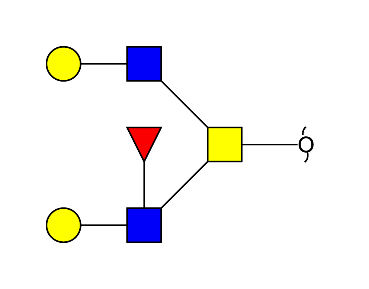 | 1098b | 1099b | 4 | 0.69±0.15 | 0.35±0.20 |
| 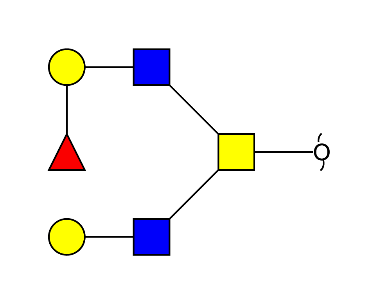 | 1098c | 1099c | 4 | 0.82±0.07 | 0.90±0.32 |
| 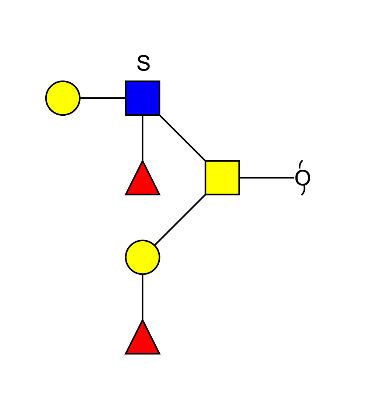 | 1121 | 1122 | 2 | 2.54±0.26 | 1.16±0.63 |
| 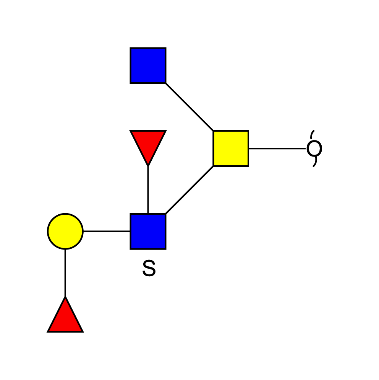 | 1162a | 1163a | 4 | 3.19±0.47 | 4.02±1.95 |
| 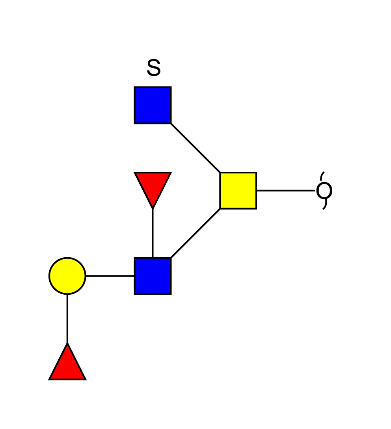 | 1162b | 1163b | 4 | 2.50±1.25 | 0.00±0.00 |
| 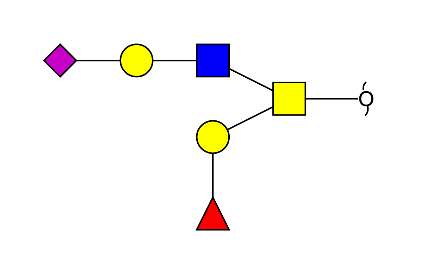 | 1186a | 1187a | 2 | 0.12±0.07 | 0.00±0.00 |
| 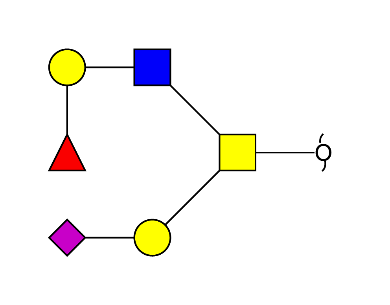 | 1186b | 1187b | 2 | 0.07±0.05 | 0.00±0.00 |

H: Healthy controls, D: Diarrheal piglets.

Values provided are in % relative abundance with the standard error.
