# Supplementary material for: Mucin O-glycan-microbiota axis orchestrates gut homeostasis in a diarrheal pig model
Source: Microbiome. 2022 Aug 31;10:139. doi: 10.1186/s40168-022-01326-8 (PMC9429786; doi:10.1186/s40168-022-01326-8)
Supplement: Supplementary file 12 — Additional file 11: Table S4. The standard of fecal rating. [file 40168_2022_1326_MOESM11_ESM.docx]

**Table S4 The standard of fecal rating**

| Score | Characteristics |
| --- | --- |
| 1 | Firm, but not hard, pliable;  Segmented in appearance;  Little or no residue on ground when picked up |
| 2 | Log shaped, moist surface;  Little or no visible segmentation;  Leaves residue on ground, but holds form when picked up |
| 3 | Very moist and soggy;  Log shaped;  Leaves residue on ground and loses form when picked up |
| 4 | Very moist but has a distinct shape;  Present in piles rather than logs;  Leaves residue on ground and loses form when picked up |
| 5 | Has texture, but no defined shape;  Present as piles or spots;  Leaves residue on ground when picked up |
| 6 | Watery;  No texture;  Present in flat puddles |
