# Supplementary material for: Mucin O-glycan-microbiota axis orchestrates gut homeostasis in a diarrheal pig model
Source: Microbiome. 2022 Aug 31;10:139. doi: 10.1186/s40168-022-01326-8 (PMC9429786; doi:10.1186/s40168-022-01326-8)
Supplement: Supplementary file 13 — Additional file 12: Table S5. The information of piglets. [file 40168_2022_1326_MOESM12_ESM.docx]

| Piglet | Litter | House | Gender | Body weight at birth/kg | Final body weight/kg | Body weight of sow/kg |
| --- | --- | --- | --- | --- | --- | --- |
| H1 | 1 | 1 | male | 2.11 | 8 | 296 |
| H2 | 2 | 2 | male | 1.9 | 8 | 268.5 |
| H3 | 3 | 3 | female | 2.18 | 8 | 284.5 |
| H4 | 4 | 4 | female | 1.88 | 6.9 | 291 |
| H5 | 4 | 4 | male | 1.95 | 7.9 | 291 |
| H6 | 5 | 5 | female | 2.06 | 7.9 | 279 |
| H7 | 5 | 5 | male | 1.77 | 7.8 | 279 |
| H8 | 6 | 6 | female | 1.87 | 7.9 | 281 |
| D1 | 1 | 1 | male | 2.06 | 7.8 | 296 |
| D2 | 2 | 2 | male | 2.05 | 7.9 | 268.5 |
| D3 | 3 | 3 | female | 2.03 | 8 | 284.5 |
| D4 | 4 | 4 | female | 1.97 | 7.5 | 291 |
| D5 | 4 | 4 | male | 2.03 | 7 | 291 |
| D6 | 5 | 5 | female | 1.9 | 6.5 | 279 |
| D7 | 5 | 5 | male | 1.87 | 7.3 | 279 |
| D8 | 6 | 6 | female | 1.81 | 7 | 281 |

**Table S5 The information of piglets**
