# Supplementary material for: Mucin O-glycan-microbiota axis orchestrates gut homeostasis in a diarrheal pig model
Source: Microbiome. 2022 Aug 31;10:139. doi: 10.1186/s40168-022-01326-8 (PMC9429786; doi:10.1186/s40168-022-01326-8)
Supplement: Supplementary file 14 — Additional file 13: Table S6. Primer sequences used for qPCR in this study. [file 40168_2022_1326_MOESM13_ESM.docx]

**Table S6 Primer sequences used for qPCR**

| Gene | Accession number | Primer sequences (5'-3') |
| --- | --- | --- |
| *β-actin* | XM_003357928.4 | F: GCGTAGCATTTGCTGCATGA |
|  |  | R: GCGTGTGTGTAACTAGGGGT |
| *GAPDH* | XM_021091114.1 | F: CGTGTCGGTTGTGGATCTGA |
|  |  | R: TGACGAAGTGGTCGTTGAGG |
| *ZO-1* | XM_005659811.1 | F: CTCCAGGCCCTTACCTTTCG |
|  |  | R: GGGGTAGGGGTCCTTCCTAT |
| *Claudin2* | NM_001161638.1 | F: GCATCATTTCCTCCCTGTT |
|  |  | R: TCTTGGCTTTGGGTGGTT |
| *Occludin* | NM_001163647.2 | F: CAGGTGCACCCTCCAGATTG |
|  |  | R: TATGTCGTTGCTGGGTGCAT |
| *Claudin4* | NM_001161637.1 | F: CAACTGCGTGGATGATGAGA |
|  |  | R: CCAGGGGATTGTAGAAGTCG |
| *MUC2* | XM_021082584.1 | F: CGCATGGATGGCTGTTTCTG |
|  |  | R: ATTGCTCGCAGTTGTTGGTG |
| *TFF3* | NM_001243483.1 | F: GGGAAAAAGCTCCCTGGCTA |
|  |  | R: TCAAGGGTCACGGAAAGTGG |
| *MUC4* | XM_021068274.1 | F: TTCACTCCAACCATCCTTCCA |
|  |  | R: CTCGTTCCACTTGTCTGTTCC |
| *RETNLB* | XM_005670236.3 | F: CTGACCAGTCTTTGCAGACCT |
|  |  | R: CACTGAGAACCCCTTGCGAT |
| *AGR2* | XM_005667660.3 | F: AGCTCCTCCCTCTGTGTTAGG |
|  |  | R: TGAGTATGTTCACCAGTGCCTT |
| *ERN1* | XM_005668695.3 | F: GCGAAGCATGTGCTGAAACA |
|  |  | R: TATCCGGTCACTCACGTCCT |
| *ERN2* | XM_021086463.1 | F: TTCTGCTCTCTCACAGGATGC |
|  |  | R: TGGTCTTGCTCTGAGGGGTT |
| *SLC26A3* | XM_021101946.1 | F: TGGGAGCAGTAGTGGTGAG |
|  |  | R: TGAATGATGCCAGAAAGAA |
| *FUT1* | NM_214068.2 | F: CCCATATCGTGCCTCTTGCT |
|  |  | R: CCAGGCCATGGGCTACATAC |
| *FUT2* | NM_214069.1 | F: CAATTACACGCTCCCGGACT |
|  |  | R: CCAGCCGACATCAGTGCTTA |
| *GAL3ST1* | XM_005670826.3 | F: AGGGACATGCCACCTGCTAT |
|  |  | R: CACTGGGAAACAGGAACGCT |
| *GAL3ST2* | XM_021074665.1 | F: GAAAGTCATGCCCAACGACAC |
|  |  | R: GGAGGACTCGAGCTGGAAGA |
| *ST6GAL1* | XM_021070035.1 | F: CACCGCAAACCCTTCGGA |
|  |  | R: ACTCTGCCTTTTAAACGTGTCTG |
| *GCNT1* | XM_021065082.1 | F: CGACAAAACACATCCTCCT |
|  |  | R: TGCCCACTCCATAAACTTC |
| *B3GNT3* | XM_003123499.4 | F: GGTTGTCTGGAGTGTTTGTGTC  R: TTTGCGATTGGTCCGAGGAG |
| *B3GALT5* | XM_021070992.1 | F: TCCAAGCAGACGTTCTTCCC |
|  |  | R: AGGTCTTGAGGCTTGACGTG |
| *B4GALT3* | XM_013996834.2 | F: GATAAGTGCCCGCGTTCCTA |
|  |  | R: GATCGCTGGGTCTGTGGAC |
| *B4GALT5* | XM_003134490.5 | F: TTGGCACAGGGTGCATAGAG |
|  |  | R: CAAACCCTTCCAGAGGGCAT |
| *B4GALT6* | XM_003127886.5 | F: TTCCGTAACCGCCATGAACA |
|  |  | R: TTGCACGGTTAAAAGGCTGC |
| *B4GALT7* | NM_001168422.1 | F: CTACCAGCTGTGCAATGGGA |
|  |  | R: GCAGGTGGCGAAAAGTCTTG |
| *TNF-α* (H) | NM_001065.4 | F: TGTGTGGCTGCAGGAAGAAC |
|  |  | R: GCAATTGAAGCACTGGAAAAGG |
| *IL-18* (H) | NM_001243211.2 | F: TGCCCTCCTGGCTGCCAACT |
|  |  | R: TCAGCAGCCATCTTTATTCCTGCG |
| *IL-6* (H) | NM_001371096.1 | F: ATCCTCGACGGCATCTC |
|  |  | R: TCAGCCATCTTTGGAAGG |
| *IL-8* (H) | NM_001354840.3 | F: GACATACTCCAAACCTTTCCA |
|  |  | R: AACTTCTCCACAACCCTCTG |
| *TLR4* (H) | NM_003266.4 | F: CTCCTGCGTGAGACCAGAAA |
|  |  | R: AATGGAATCGGGGTGAAGGG |
| *TLR5* (H) | NM_003268.6 | F: GACCCTCTGCCCCTAGAATAA |
|  |  | R: GCCATGAGCACCACTCCTA |
| *NF-κB* (H) | NM_001382627.1 | F: TGGGAATGGTGAGGTCACTCT |
|  |  | R: TCCTGAACTCCAGCACTCTCTTC |
| *AGR2* (H) | XM_005249581.5 | F: GACTCACACAAGGCAGGTGG |
|  |  | R: ATGAGTTGGTCACCCCAACC |
| *BiP* (H) | NM_005347.5 | F: TGTTCAACCAATTATCAGCAAACTC |
|  |  | R: TTCTGCTGTATCCTCTTCACCAGT |
| *ERN1* (H) | NM_001433.5 | F: CGGCCTTTGCAGATAGTCTC |
|  |  | R: ACGTCCCCAGATTCACTG |
| *ZO-1* (H) | NM_001355014.2 | F: CGTCACCTACCACCTCGT |
|  |  | R: GCCTTCTCCCACTCTGTCT |
| *Occludin* (H) | NM_002538.4 | F: CATTGCCATCTTTGCCTGTG |
|  |  | R: AGCCATAACCATAGCCATAGC |
| *Claudin1* (H) | NM_021101.5 | F: CCCAGTCAATGCCAGGTACG |
|  |  | R: GGGCCTTGGTGTTGGGTAAG |
| *MUC2* (H) | NM_002457.4 | F: AAGACGGCACCTACCTCG |
|  |  | R: TTGGAGGAATAAACTGGAGAACC |
| *MUC5AC* (H) | NM_001304359.2 | F: GTTTGACGGGAAGCAATACA |
|  |  | R: CGATGATGAAGAAGGTTGAGG |
| *MUC17* (H) | NM_001040105.2 | F: GGGCCAGCATAGCTTCGA |
|  |  | R: GCTACAGGAATTGTGGGAGTTCA |
| *TFF3* (H) | NM_003226.4 | F: CCAAGGACAGGGTGGACTG |
|  |  | R: AAGGTGCATTCTGCTTCCTG |
| *RETNLB* (H) | NM_032579.2 | F: CACCCAGGAGCTCAGAGATCTAA |
|  |  | R: ACGGCCCCATCCTGTACA |
| *FCGBP* (H) | NM_003890.2 | F: AGGAGTGCCGGTTAGAGGAT |
|  |  | R: AGACATAGGAGCAGGAGCCA |
| *ATF4* (H) | NM_001675.4 | F: GTTCTCCAGCGACAAGGCTA |
|  |  | R: ATCCTGCTTGCTGTTGTTGG |
| *ATG5* (H) | NM_001286108.2 | F: ATTCCAACTTGTTTCACGCTAT |
|  |  | R: AAATAGTATGGTTCTGCTTCCCT |
| *ATG7* (H) | NM_001349234.2 | F: ACGATCGGATGAATGAGCCT |
|  |  | R: AGATCTCAGCAGCTTGGGTT |
| *LC3B* (H) | NM_022818.5 | F: AGATTGGTGTGGAGACGCTG |
|  |  | R: AGCAGCTTCCTGTTCTGGAT |
| *P62* (H) | NM_001142298.2 | F: CATCGGAGGATCCGAGTGTG |
|  |  | R: AGATGTGGGTACAAGGCAGC |
| *E. coli* | MZ749737.1 | F: GTTAATACCTTTGCTCATTGA |
| *16S* | EUB338/518 | R: ACCAGGGTATCTAATCCTGTT  F: ACTCCTACGGGAGGCAGCAG  R: ATTACCGCGGCTGCTGG |
